# Riverscape heterogeneity shapes population diversity for a migratory fish

**DOI:** 10.1002/eap.70247

**Published:** 2026-06-05

**Authors:** Jeffrey R. Baldock, William C. Rosenthal, Robert K. Al‐Chokhachy, Matthew R. Campbell, Catherine E. Wagner, Annika Walters

**Affiliations:** ^1^ Wyoming Cooperative Fish and Wildlife Research Unit, Department of Zoology and Physiology and Program in Ecology and Evolution University of Wyoming Laramie Wyoming USA; ^2^ Department of Botany and Program in Ecology and Evolution University of Wyoming Laramie Wyoming USA; ^3^ U.S. Geological Survey Northern Rocky Mountain Science Center Bozeman Montana USA; ^4^ Eagle Fish Genetics Laboratory, Idaho Department of Fish and Game Eagle Idaho USA; ^5^ U.S. Geological Survey, Wyoming Cooperative Fish and Wildlife Research Unit, Department of Zoology and Physiology and Program in Ecology and Evolution University of Wyoming Laramie Wyoming USA; ^6^ Present address: Department of Fisheries, Wildlife, and Conservation Sciences Oregon State University Corvallis Oregon USA

**Keywords:** connectivity, genetic stock identification, groundwater, headwater streams, large rivers, life history diversity, metapopulation theory, migration, source–sink dynamics, spatial structure

## Abstract

Habitat patch dynamics can scale up to influence population demography and diversity with implications for resilience to environmental stochasticity. But how the spatial arrangement and size of habitat patches interact with other components of habitat heterogeneity to shape population diversity at larger spatial scales is not well understood. For riverine fishes, there is increasing evidence that tributary streams provide critical demographic support to main stem rivers. However, the extent to which main stem rivers rely on demographic contributions from tributaries, and the factors underlying this dependence, have not been assessed. Here, we used genetic stock identification to evaluate the effect of tributaries on population diversity of Yellowstone cutthroat trout (*Oncorhynchus virginalis bouvieri*) occupying the main stem Snake River, Wyoming, USA. We found that the main stem relied almost entirely on tributaries for demographic support, but main stem composition varied spatially among river sections. Distance between habitat patches, catchment area, and groundwater availability acted in concert to determine the contribution of specific tributaries to the main stem, but contributions were ultimately modulated by habitat connectivity. We also found evidence for multi‐scale spatial structure in tributary contributions, providing insight into untested drivers of main stem river population diversity. Our results demonstrate how spatially discrete and distributed riverscape attributes influence population diversity at broader spatial scales, illustrating how ecosystem resilience emerges from the dynamic, two‐way exchange of individuals and energy across habitat networks. Management plans for large rivers that address the ecological contributions of tributaries may be needed to achieve optimal outcomes. Similarly, conservation strategies that exclusively focus on headwater streams may fail to capture the broader habitat requirements necessary to maintain robust cold‐water fish populations and associated recreational fisheries, particularly under global environmental change.

## INTRODUCTION

The effects of habitat patch dynamics on population demography at larger spatial scales are critically important as population diversity confers resilience to environmental stochasticity (Luck et al., 2003; Schindler et al., 2010). Population diversity arises in spatially heterogeneous environments where local adaptation and natal site fidelity have led to distinct evolutionary units that respond differently to shared stressors (Elmqvist et al., 2003; Kawecki & Ebert, 2004). Habitat heterogeneity can affect habitat‐specific demographic rates, influencing population growth and among‐patch dispersal (Levins, 1969; Pulliam, 1988). But population dynamics also depend on the size and spatial arrangement of source habitats (Hanski, 1998; Wilson et al., 2023), which may interact with local environmental conditions and movement barriers to affect population diversity at larger spatial scales (Campbell Grant et al., 2007; Fagan, 2002; Moilanen & Hanski, 1998). Although this theoretical framework guides much of modern conservation science (e.g., Anderson et al., 2015), empirical support for how population diversity arises from spatially structured, heterogeneous landscapes is lacking.

In river networks, habitat patches are arranged hierarchically from small headwater tributaries to larger main stem rivers, and habitat conditions vary across both space and time (Frissell et al., 1986; Stanford et al., 2005). For riverine fishes, tributary streams often provide important spawning and juvenile rearing habitat (Healy & Smith, 2023; Schlosser, 1991), allowing for natal site fidelity that can generate local adaptation and response diversity (Braun et al., 2016; Quinn, 2018). However, limited habitat availability and short growing seasons can constrain fish growth and production in tributaries, prompting seasonal migration to main stem rivers where larger body sizes can be attained due to warmer temperatures and increased food availability (Al‐Chokhachy et al., 2022; Armstrong et al., 2021; Petty et al., 2014). Therefore, tributaries may provide key demographic support to main stem rivers (Healy & Smith, 2023; Tsuboi et al., 2022), where individuals from genetically distinct populations mix. Nonetheless, how demographic contributions to main stem rivers vary with tributary size and location, local habitat conditions, and movement barriers has not been tested.

The relative importance of tributary source habitats to main stem rivers may reflect the effects of tributary habitat characteristics on fish production. For example, in runoff and snowmelt‐dominated streams, water temperature and flow vary considerably over time and recruitment is often limited by suboptimal environmental conditions (Coleman & Fausch, 2007). In contrast, groundwater discharge to streams stabilizes temperature and flow regimes, extending the growing season and leading to high juvenile recruitment where individual growth is limited by density dependence (Baldock et al., 2025; Matte et al., 2020). Density‐dependent resource limitation may increase dispersal rates as fish seek to reduce physiological stress (Einum et al., 2006; Forseth et al., 1999). Therefore, highly productive tributary habitats, such as those fed by groundwater, may contribute disproportionately (relative to their size) to main stem rivers via density‐dependent spillover (sensu Abesamis & Russ, 2005).

Although the spatial configuration of tributary habitat conditions can influence contributions to main stem river fish populations, these relationships may ultimately be filtered by habitat fragmentation and connectivity. Both natural and anthropogenic landscape features can restrict fish movement, gene flow, and the expression of migratory life histories (Kanno et al., 2014; White et al., 2020). Assessing the impacts of barriers on tributary support of main stem populations is important because habitat fragmentation can increase extinction risk (Fagan, 2002; Letcher et al., 2007). How habitat connectivity and fragmentation mediate the role of tributary characteristics in supporting main stem river fish populations is not well understood but is essential for directing species conservation and habitat restoration efforts.

**FIGURE 1:**
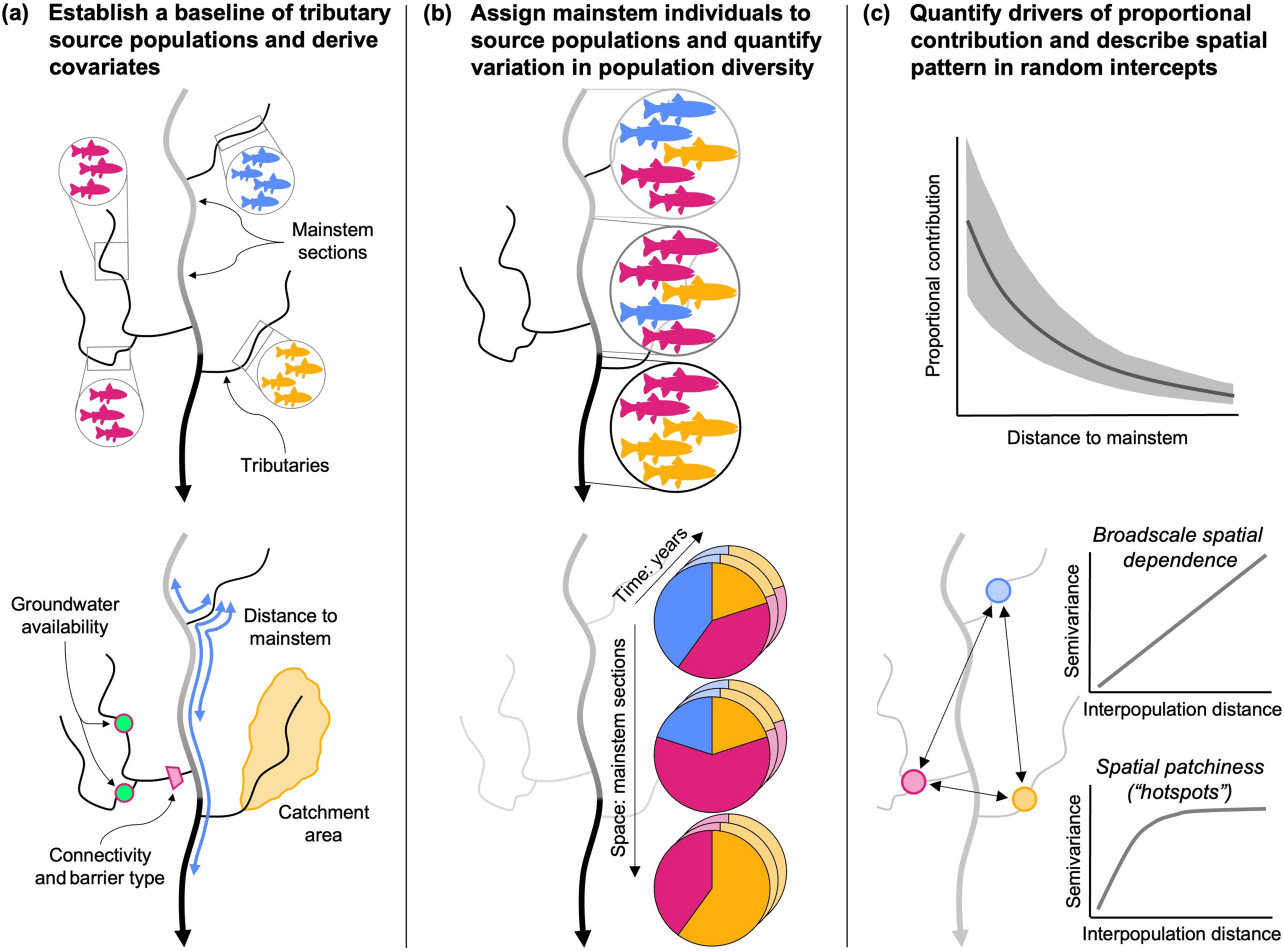
Conceptual diagram illustrating the methodology for sampling and analysis. (a) We established a baseline of genetically distinct source populations (colors) using juvenile trout collected from tributary streams. For each source population, we derived covariates representing flow line distance to each main stem river section, catchment area, groundwater availability, and connectivity to the main stem. (b) We used genetic stock identification to assign individuals sampled in the main stem Snake River to source populations based on genetic similarity to the baseline and quantified spatial (among section) and temporal (among year) variation in population diversity (i.e., aggregate representation of source populations). (c) We quantified the effects of source population covariates on proportional contribution to the main stem using hierarchical regression and described spatial patterns in random intercepts (i.e., variation in tributary contributions not explained by the covariates) using empirical semivariograms. All illustrations by Jeffrey R. Baldock.

In this study, we used genetic stock identification (Figure 1) to evaluate the contribution of tributary streams to a main stem population of native cold‐water fish occupying a large riverscape: Yellowstone cutthroat trout (*Oncorhynchus virginalis bouvieri*) in the upper Snake River watershed, Wyoming, USA. Genetic stock identification assigns individuals captured in a common environment (e.g., the main stem) to contributing stocks based on genetic similarity to groups of individuals captured from known locations (e.g., tributary source populations). We assessed the degree to which main stem population composition (i.e., population diversity) varied across space and time and quantified the effect of tributary characteristics (distance to the main stem, catchment size, groundwater availability, and connectivity) on demographic contributions to the main stem. Our results provide insight into the effects of riverscape structure on functional connectivity between tributary and main stem habitats for an ecologically and economically important riverine fish. How the spatial arrangement and size of habitat patches interact with local environmental conditions and dispersal barriers to affect population diversity at larger spatial scales has important implications for riverscape conservation in the context of global climate and land‐use change.

## METHODS

### Study system and species

**FIGURE 2:**
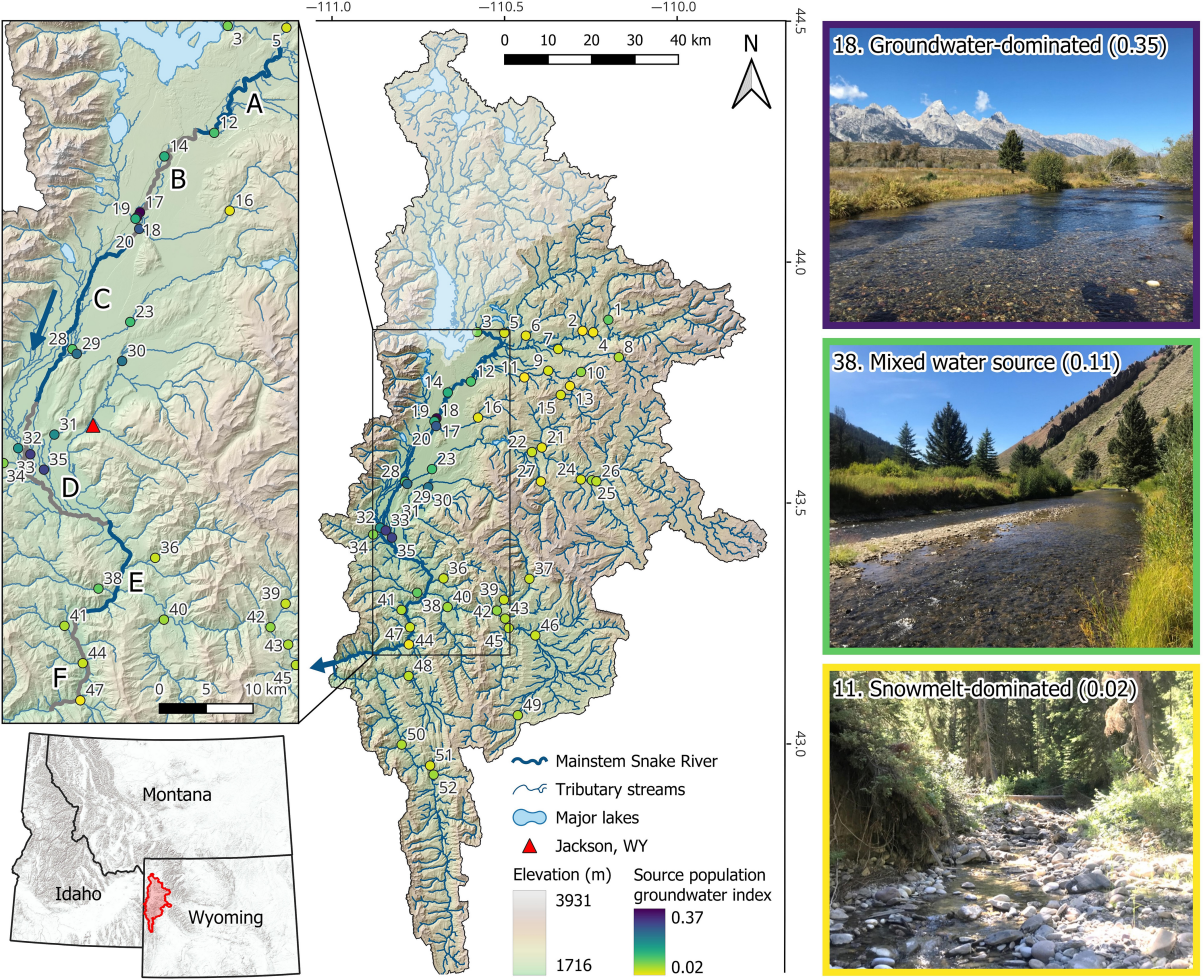
Map of the upper Snake River watershed. Thick and thin flow lines represent the main stem Snake River and tributary streams, respectively. Points mark sampling locations for source populations, numbered as in Appendix S1: Table S1; color denotes the groundwater index. Flow direction is denoted by the blue arrows. Opaque region in the northern portion of the watershed was excluded from our study as it is upstream of Jackson Lake Dam, a complete barrier to fish passage. Main stem sampling sections are depicted in the inset map (alternating colors and lettered as in Figure 4). Red triangle marks the town of Jackson, Wyoming, and the red outline in the regional map marks the area of detail. Regional base map provided by Esri (Redlands, CA, USA). Photos illustrate the range of stream habitat conditions among source populations, from low‐gradient groundwater‐fed streams (top) to high‐gradient snowmelt‐dominated headwaters (bottom). Numbers in parentheses and border color signify the groundwater index. Photographs courtesy of the Wyoming Cooperative Fish and Wildlife Research Unit.

We conducted our study within the upper Snake River watershed in northwest Wyoming, USA, a 10,108‐km^2^ region with elevations ranging from 1708 to 4194 m comprising the core of the Greater Yellowstone Area (Figure 2; Baldock et al., 2025). Our study area includes the stream network upstream of Palisades Reservoir, excluding the Salt River sub‐watershed (previous work found no evidence of fish movement between the Snake and Salt rivers; Homel et al., 2015) and the area upstream of Jackson Lake Dam, which forms a complete barrier to fish passage. Streamflow in the Snake River and many tributaries is sourced primarily from snowmelt, while shallow groundwater discharge constitutes the majority of flow in other tributaries (Hostetler et al., 2021). Thus, streams fall along a hydrologic gradient of groundwater availability, with important effects on habitat conditions (e.g., stream temperature) that mediate juvenile trout growth and production (Baldock et al., 2025).

Yellowstone cutthroat trout (hereafter, trout) are a regionally important component of aquatic and terrestrial food webs and support economically and culturally valuable recreational fisheries (Gresswell, 2011; Koel et al., 2019). Habitat degradation and hybridization and competition with non‐native species have led to widespread declines (Gresswell, 2011) and anticipated climate warming further threatens many extant populations (Wenger et al., 2011). However, the upper Snake River watershed remains a stronghold and conservation priority for this species as both resident and migratory life history forms are maintained (Al‐Chokhachy et al., 2018; Haak & Williams, 2012), with migrants moving between tributaries for spawning and larger main stem rivers during nonreproductive periods (Homel et al., 2015).

### Fish sampling

Genetic stock identification requires two types of samples: “baseline” samples which characterize genetic variation within and among source populations and “mixture” samples collected from a common environment, whose stock composition is unknown. Between 2020 and 2022, we collected trout tissue samples from tributary streams to establish a baseline of potential source populations (Figure 1a; Hargrove et al., 2023). We captured fish using either dip‐netting or backpack electrofishing. We primarily sampled age‐0 individuals (<60 mm total length), which possess limited swimming abilities and therefore likely represent offspring from the local spawning population. When age‐0 fish were not found, we sampled individuals up to 200 mm. We aimed to sample 30–40 individuals per location because these sample sizes allow for accurate genetic assignments when source populations are well differentiated (*F*_ST_ > 0.01; Beacham et al., 2020). Previous research on trout in our study area indicated that populations exhibited a global *F*_ST_ of 0.04 (Kovach et al., 2011).

We used raft electrofishing to collect tissue samples from adult trout in the main stem Snake River, representing mixture samples for genetic stock identification (Figure 1b). Sampling occurred annually in mid‐October from 2020 to 2022, following flow reductions from Jackson Lake Dam (to maximize capture efficiency) and trout settlement into overwintering habitat (Homel et al., 2015). We sampled adult trout from six sections of the Snake River to evaluate spatiotemporal variability in population composition (Figure 2). Section A was not sampled in 2020 because the sole access point was closed at that time. We sampled approximately 500 adult trout annually, with samples proportionally distributed among the six river sections according to section length. We dispersed collections spatially within each section using intermittent electrofishing. We also distributed collections evenly across six body‐size categories (total length in mm): 201–250, 251–300, 301–350, 351–400, 401–450, and ≥450 (Meyer et al., 2003). Because smaller fish numerically dominate the population (Wyoming Game and Fish Department, 2019), it was necessary to sample size classes disproportionately to accurately estimate contributions of larger, less abundant individuals, which are important for key population processes such as spawning and fecundity.

### Genotyping

We genotyped all tissue samples following standard protocols, briefly summarized here. We extracted genomic DNA from fin‐clip samples using the Nexttec Genomic DNA Isolation Kit (XpressBio, Thurmont, Maryland, USA) following the manufacturer's protocol. We genotyped individual fish using the genotyping‐in‐thousands by sequencing protocol (GT‐seq; Campbell et al., 2015). We then screened samples with a panel of 353 single nucleotide polymorphic (SNP) loci, including 38 previously developed species‐diagnostic SNPs for assessing hybridization with rainbow trout (Pritchard et al., 2013). We organized genotypes using the R package *EFGLmh* (https://github.com/delomast/EFGLmh/). This package generates formatted inputs for analytical software, including GenAlEx, a program used for population genetic analyses of codominant, haploid, and binary genetic data (Peakall & Smouse, 2006). Two samples were identified as hybrids with rainbow trout (*Oncorhynchus mykiss*) and removed from analyses. We removed species‐diagnostic, invariant, and mitochondrial DNA markers, leaving 266 for analyses. At the time of publication, marker information for these 266 SNP loci, including primer sequences (forward and reverse), was not available from the Idaho Department of Fish and Game. This information is available upon request. We retained only those samples that were genotyped at ≥85% of loci.

### Baseline source populations

We estimated relatedness among individuals within baseline collections using the R package *demerelate* to identify close relatives (i.e., full siblings) as their inclusion can bias genetic stock identification (Kraemer & Gerlach, 2017; Östergren et al., 2020; R Core Team, 2025). We classified pairs of individuals with a Wang relatedness coefficient >0.4 as full siblings (Källo et al., 2023). Following Östergren et al. (2020), we retained two individuals per full‐sibling family to balance allele frequency estimation accuracy and sample size.

We aggregated baseline collections into reporting units representing tributary source populations (hereafter, source populations; Figure 1a) through an iterative process based on assignment accuracy, geographic proximity, and indices of pairwise genetic differentiation (*F*_ST_). We aimed to minimize aggregation to best estimate tributary‐specific contributions, while ensuring sufficient assignment accuracy. We first conducted preliminary leave‐one‐out self‐assignment tests using the “self_assign” function in the R package *rubias* (Moran & Anderson, 2019). We then used confusion matrices to identify collections with low self‐assignment rates (<0.7) and their associated receiving collections. We also calculated pairwise *F*_ST_ (Weir & Cockerham, 1984) among collections to identify sets of collections that may function as a single genetic population. Comparing the confusion matrices and pairwise *F*_ST_ values indicated that self‐assignment errors primarily occurred among geographically proximate collections that were poorly differentiated (*F*_ST_ < 0.01). We thus aggregated collections into source populations if self‐assignment confusion among collections was high, pairwise *F*_ST_ was low (<0.01; sensu Araujo et al., 2014), and the collections were made at different locations along the same tributary stream. We excluded six collections from our baseline due to small sample sizes (*n* ≤ 5 per collection) and because aggregation was not feasible. Excluding these collections did not affect assignment accuracy.

We tested the accuracy and precision of our final baseline source populations by simulating main stem Snake River mixtures using baseline allele frequencies following the leave‐one‐out approach of Anderson et al. (2008). We used the “assess_reference_loo” function in *rubias* to simulate 500 mixtures of 1000 individuals and assigned those individuals back to source populations (Hargrove et al., 2023). We then calculated residuals (the difference between the true and estimated mixing proportions for each source population and simulation) and compared the distribution of residuals to 0 to assess bias in estimated contributions for each source population independently.

### Mixture analysis

We quantified the contribution of source populations to the main stem Snake River using the “infer_mixture” function in *rubias* (Figure 1b). We treated each sampling event (over 3 years and six sections of the main stem Snake River) as separate mixtures to evaluate spatiotemporal variation in tributary contributions. *Rubias* functions in a Bayesian probabilistic framework, whereby each mixture sample (i.e., adult trout) assigns to every source population with a given probability. Source population mixing proportions are then calculated by summing probabilities across all individuals. To account for bias in mixing proportions (as estimated above), we generated bootstrap‐corrected mixing proportions using the leave‐one‐out approach implemented within the “infer_mixture” function (Moran & Anderson, 2019). We computed *z*‐scores as an additional check on assignment accuracy, which represent how similar an individual is to samples in the baseline dataset. We considered individuals with *z*‐scores two SDs beyond the mean to have originated from source populations missing from our baseline (Bowersox et al., 2023).

### Tributary covariates

For each source population, we derived covariates hypothesized to affect contributions to the main stem: distance to each main stem river section, catchment area, groundwater availability, and connectivity (Figure 1a). We thus required a stream network spatial object that accurately represented flow lines in the study area, particularly in areas where groundwater upwelling creates dense drainage networks where many of our collections were made. We used the WhiteboxTools library implemented in R (Lindsay, 2016; Wu & Brown, 2022) to delineate a stream network from a 1/3 arc‐sec (~10 m) bare‐earth digital elevation model (accessed November 15, 2023, https://apps.nationalmap.gov/downloader/) using a threshold flow accumulation value of 25,000 cells (2.5 km^2^). This approach tended to omit flow lines for streams originating from high‐volume groundwater springs, as these catchments are often small and poorly defined (Whiting & Moog, 2001). We manually delineated flow lines for these streams based on our knowledge of the study area and merged these features to the stream network using ArcMap ver. 10.8 (Esri, Redlands, CA). Refer to Baldock et al. (2025) for detailed methodology.

We used WhiteboxTools to delineate catchments and calculate catchment area for each source population. For source populations composed of multiple collections, we delineated catchments for the collection located furthest downstream and interpreted catchment area as a metric of stream habitat availability. We used the *riverdist* package in R (Tyers, 2024) to calculate pairwise flow line distance between each source population (the furthest downstream sampling location) and the midpoint of each section of the main stem Snake River.

We derived an index of groundwater availability for each source population following Baldock et al. (2025). We used MaxEnt version 3.4.4 (Elith et al., 2011; Phillips & Dudík, 2008; https://biodiversityinformatics.amnh.org/open_source/maxent/, accessed on 17 July 2023) to predict groundwater spring prevalence using 219 spring locations as presence points and 11 geologic and topographic layers as predictors (Gerlach et al., 2022). MaxEnt outputs a raster of spring prevalence (i.e., the probability that each cell contains a groundwater spring), from which we calculated the inverse distance‐weighted catchment mean to describe groundwater availability for each source population. Refer to Baldock et al. (2025) for detailed methodology. Relative to the prior study, we extended the groundwater model to include the Greys River basin, where some of our source populations were located. High groundwater index values indicate reaches where water is sourced primarily from shallow groundwater aquifers and streamflow and temperature regimes exhibit limited seasonal variation (Baldock et al., 2025). Low values indicate reaches where water is sourced from snowmelt and flow and temperature regimes are more variable.

We assessed connectivity between source populations and the main stem Snake River based on field observations and the National Aquatic Barrier Inventory and Prioritization Tool (https://aquaticbarriers.org/, accessed 10 July 2024). We treated connectivity as a categorical variable with one of four values: connected, culvert/diversion, low flow, or waterfall. “Connected” source populations (*n* = 39) are those with no barriers to fish passage along the river corridor between baseline collections and the main stem. “Culvert/diversion” barriers are locations where fish passage is partially or completely blocked by an impassable culvert or irrigation diversion. Culverts and diversion dams were aggregated into a single classification due to low sample sizes (*n* = 2 and 1 source populations, respectively). “Low flow” barriers are defined by reaches with intermittent streamflow, typically occurring between July and October (*n* = 7). “Waterfall” barriers are cascades of at least 1 m in height (*n* = 3).

### Statistical analyses

#### Spatiotemporal variation in population diversity

We used zero‐and‐one inflated Dirichlet regression implemented in the R package *zoid* to understand spatiotemporal variation in the composition (i.e., population diversity) of the main stem Snake River population of trout (Jensen et al., 2022; Figure 1b). We tested five alternative hypotheses (Table 1): main stem composition is (1) constant over space and time (null), (2) varies by section, (3) varies by year, (4) varies by section and year, and (5) varies by section and year, but certain sections vary more among years than others. We fit candidate models representing each hypothesis and compared models using leave‐one‐out cross‐validation (Vehtari et al., 2017). We ran models with flat Dirichlet priors, three Markov chain Monte Carlo (MCMC) chains, and 2000 burn‐in and evaluation iterations per chain.

**TABLE 1:** Summary of zero‐and‐one inflated Dirichlet regression model selection results investigating spatiotemporal variability in proportional contribution of source populations to the main stem Snake River.

ID	Model	elpd_loo_	SE (elpd_loo_)	elpd_diff_	SE (elpd_diff_)
2	Section	−1199.5	53.6	0.0	0.0
4	Section + Year	‐1228.0	51.5	−28.5	16.1
5	Section + Year + Section × Year	−1294.5	47.6	−95.0	25.3
3	Year	−1807.4	98.1	−607.9	73.7
1	Null	−3272.9	137.0	−2073.4	128.3

*Note*: ID refers to the numbered hypotheses outlined in the text. Model represents the linear predictor. Leave‐one‐out cross‐validation diagnostics are defined as follows: elpdloo = expected log pointwise predictive density, SE (elpdloo) = SE of elpdloo, elpddiff = estimated difference of elpdloo of the candidate model and top model, and SE (elpddiff) = SE of elpddiff. Candidate models are ordered according to minimum elpdloo. Models are listed in order of leave‐one‐out cross‐validation support.

#### Drivers of tributary contributions

We quantified the effects of tributary covariates on proportional contribution to the main stem Snake River using zero‐inflated beta regression (Douma & Weedon, 2019), implemented in a Bayesian framework using the R package *brms* (Bürkner, 2017; Figure 1c). We assumed proportional contribution of trout $p_{\mathrm{rsy}}$ from source population *r* to section *s* in year *y* follows a piecewise distribution when $p_{\mathrm{rsy}}$ has inflation at 0:

(1) $$f\left(p_{\mathrm{rsy}}|\omega_{\mathrm{rsy}},\mu_{\mathrm{rsy}},\phi_{\mathrm{rsy}}\right)=\left\{\begin{array}{c} \omega_{\mathrm{rsy}}\;\;\;\;\;\;\;\;\;\;\;\;\;\;\;\;\;\;\;\;\;\;\;\;\;\;\;\;\;\;\;\;\;\;\;\;\;\;\;\;\;\;\;\;\;\;\;\;\;\;\;\text{if}\;p_{\mathrm{rsy}}=0\;\\ \left(1-\omega_{\mathrm{rsy}}\right)\;\text{Beta}\left(\mu_{\mathrm{rsy}},\phi_{\mathrm{rsy}}\right)\;\;\;\;\;\;\text{if}\;p_{\mathrm{rsy}}\in \left(0,1\right) \end{array}\right.$$

where $\omega_{\mathrm{rsy}}$ is the probability of observing zero contribution and $\mu_{\mathrm{rsy}}$ and $\phi_{\mathrm{rsy}}$ are the mean and precision of the beta distribution. We modeled $\mu_{rsy}$ as a linear function of distance between each source population and the respective main stem section $D_{rs}$, catchment area $A_{r}$, groundwater index $G_{r}$, and connectivity $C_{r}$ (categorical variable) using the logit link:

(2) $$\text{logit}\left(\mu_{\mathrm{ysr}}\right)=\alpha^{\prime }+\alpha_{s}^{\prime }+\alpha_{r}^{\prime }+\beta_{1}^{\prime }D_{rs}+\beta_{2}^{\prime }A_{r}+\beta_{3s}^{\prime }G_{r}+\beta_{4}^{\prime }C_{r}$$

where $\alpha^{\prime }$ is the global intercept; $\alpha_{s}^{\prime }$ and $\alpha_{r}^{\prime }$ are offsets for section and source population (i.e., random intercepts); and $\beta_{1}^{\prime }$, $\beta_{2}^{\prime }$, $\beta_{3s}^{\prime }$, and $\beta_{4}^{\prime }$ are coefficients (i.e., slopes). We allowed the effect of groundwater $\beta_{3s}^{\prime }$ to vary by section as the spatial distribution of groundwater‐fed tributaries was nonrandom and we therefore expected greater contributions from these source populations in nearby sections. We did not include a random intercept for year as this was not supported following the results of objective 1 (refer to *Results*: *Spatiotemporal variation in population diversity*) and led to poor mixing of MCMC chains.

We modeled $\omega_{\mathrm{rsy}}$ and $\phi_{\mathrm{rsy}}$ as linear functions of covariates using the logit and log link functions, respectively:

(3) $$\text{logit}\left(\omega_{\mathrm{rsy}}\right)=\alpha^{\prime \prime }+\beta_{1}^{\prime \prime }D_{rs}+\beta_{2}^{\prime \prime }A_{r}+\beta_{3}^{\prime \prime }G_{r}+\beta_{4}^{\prime \prime }C_{r}$$

(4) $$\log\left(\phi_{rsy}\right)=\alpha^{\prime \prime \prime }+\beta_{1}^{\prime \prime \prime }D_{rs}+\beta_{2}^{\prime \prime \prime }A_{r}+\beta_{3}^{\prime \prime \prime }G_{r}+\beta_{4}^{\prime \prime \prime }C_{r}$$

where the α and β terms are intercepts and slopes, respectively, and all other terms are defined as above. We did not include random effects for $\omega_{\mathrm{rsy}}$ and $\phi_{\mathrm{rsy}}$ as a preliminary model that included random effects performed considerably worse based on posterior predictive checks, Bayesian *p* values, and Pareto *k* parameter diagnostics (Gelman, 2003; Vehtari et al., 2017). However, estimated covariate effects were very similar between models, indicating that excluding random effects had little effect on model inference.

We ran the *brms* model with uninformative priors, four MCMC chains, and 2000 burn‐in and evaluation iterations per chain. We assessed model convergence based on large effective sample sizes, potential scale reduction factors ($\hat{R}$) <1.01 for all parameters, and visual inspection of MCMC trace plots to ensure sufficient mixing of chains (Gelman & Hill, 2007). We used posterior predictive checks to evaluate goodness of fit (Gelman, 2003). We plotted model predictions against observed data to assess the ability of the model to reproduce the original dataset. We also compared the distribution of predictions to that of the observed data and inspected distributions of model residuals to ensure normality around 0.

#### Spatial pattern in random intercepts

Inspection of the beta regression model output suggested spatial autocorrelation in random intercepts, $\alpha_{r}^{\prime }$: mean source population contribution after accounting for the effects of covariates (refer to *Results*: *Spatial structure in mean contribution*). We acknowledge that spatial autocorrelation violates assumptions of random intercepts as independent and identically distributed, but our analytical approach prohibited us from modeling the spatial pattern directly. Instead, we used empirical semivariograms to describe spatial structure in random intercepts over Euclidean and watercourse distances (Peterson et al., 2013; Figure 1c). We evaluated empirical semivariograms visually to identify broadscale, fine scale, or nested heterogeneity in random intercepts (McGuire et al., 2014). Linear relationships between semivariance and distance signify spatial dependence at broad scales (e.g., latitudinal gradients), whereas inflection points signify finer scale patchiness or hotspots. Combinations of these patterns (inflection points at short distances and linear relationships over longer distances) signify nested heterogeneity: hotspots superimposed over a gradient. Differences in semivariograms based on Euclidean versus watercourse distances provide insight into whether the drivers of spatial patterns are linked to regional landscape factors or the geometry of the stream network, respectively. We calculated semivariance over 20 distance bins using the R package *SSN2* (Dumelle et al., 2024). Calculations were based on ≥21 pairs of points, except for the smallest distance category, which was based on 16 and 17 pairs for Euclidean (0.0–2.8 km) and watercourse (0.0–5.9 km) distances, respectively. In addition to empirical semivariograms, we qualitatively described spatial structure by visually examining the distribution of random intercepts across eight geographic regions approximated by HUC‐10 (hydrologic unit code) watersheds and by latitude.

## RESULTS

### Baseline and mixture samples

After filtering, our baseline consisted of 1710 juvenile trout representing 63 collections within the upper Snake River watershed. We aggregated collections into 52 source populations with 1–3 collections and 10–82 individuals per population (Appendix S1: Table S1). Self‐assignment rates for our final set of source populations ranged from 0.48 to 1.00 (weighted mean = 0.84, Appendix S1: Figure S1). We found no relationship between self‐assignment rate and source population sample size. Generally, there was strong genetic differentiation among source populations: 99.7% of pairwise *F*_ST_ values exceeded 0.01 (range = 0.006–0.293; Appendix S1: Figure S2). Results from the mixture simulation indicated little bias in estimated contributions (maximum mean bias = 1.7%) and SDs for 40 (out of 52) source populations overlapped 0, suggesting high assignment accuracy for most populations (Appendix S1: Figure S3).

We retained 1507 adult trout genotypes for genetic stock identification (*n* = 487, 508, and 512 for years 2020, 2021, and 2022, respectively). Bootstrap‐corrected mixing proportions closely approximated uncorrected mixing proportions (Pearson's *r* = 0.99; Appendix S1: Figure S4), indicating source populations were well characterized. Fish sampled in the main stem Snake River assigned to source populations with high certainty: 82.5% of individuals assigned to a single‐source population with >70% probability. Additionally, median assignment probability was <70% in four source populations, but only one individual was assigned to each (4 of 1507 total adult trout), suggesting modeling results were likely robust to uncertainty in genetic assignments at both the individual and population levels. Furthermore, individual *z*‐scores were normally distributed (Appendix S1: Figure S5), indicating our baseline dataset was largely complete with respect to the source populations contributing fish to the main stem. However, 6.5% of fish had *z*‐scores two SDs beyond the mean, suggesting these individuals originated from source populations not included in our baseline. Missing source populations did not appear to affect proportional contribution estimates as we found no relationship between individual *z*‐scores and assignment probabilities.

**FIGURE 3:**
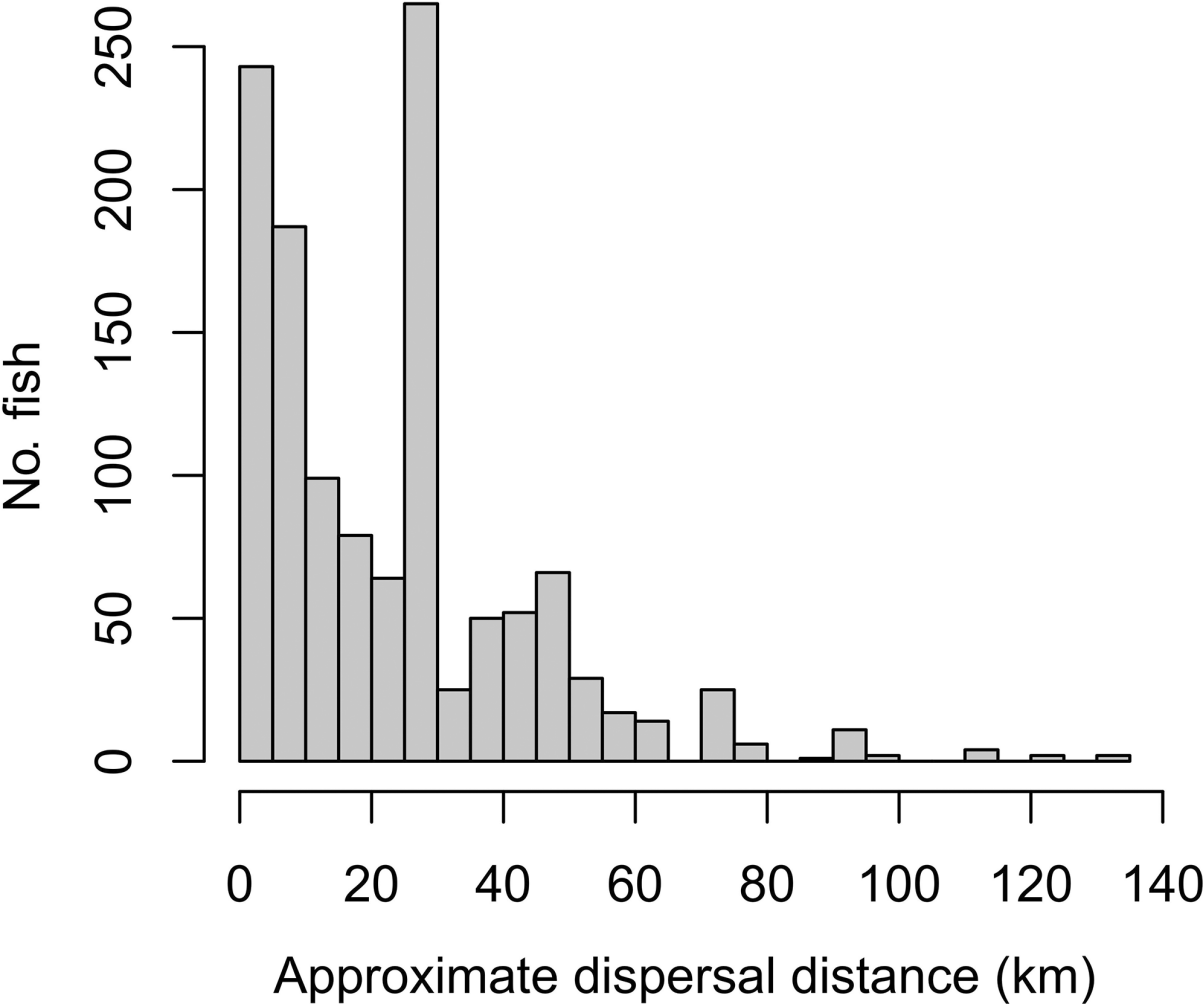
Distribution of approximate dispersal distance (i.e., flow line distance between the midpoint of the main stem section of capture and the source population of origin) for adult trout that assigned to a single source population with >70% probability. (The peak at 25–30 km is primarily driven by the large contribution of source population 32 to section D of the Snake River.)

Of the adult trout assigned to a single source population with >70% probability, approximate dispersal distance (flow line distance between the midpoint of the main stem section of capture and the source population of origin) was strongly right skewed (Figure 3). Most fish dispersed relatively short distances (median = 24 km), but some fish traveled as far as 134 km. Results were similar when calculated for fish assigning to a single source population with >95% probability.

### Spatiotemporal variation population diversity

**FIGURE 4:**
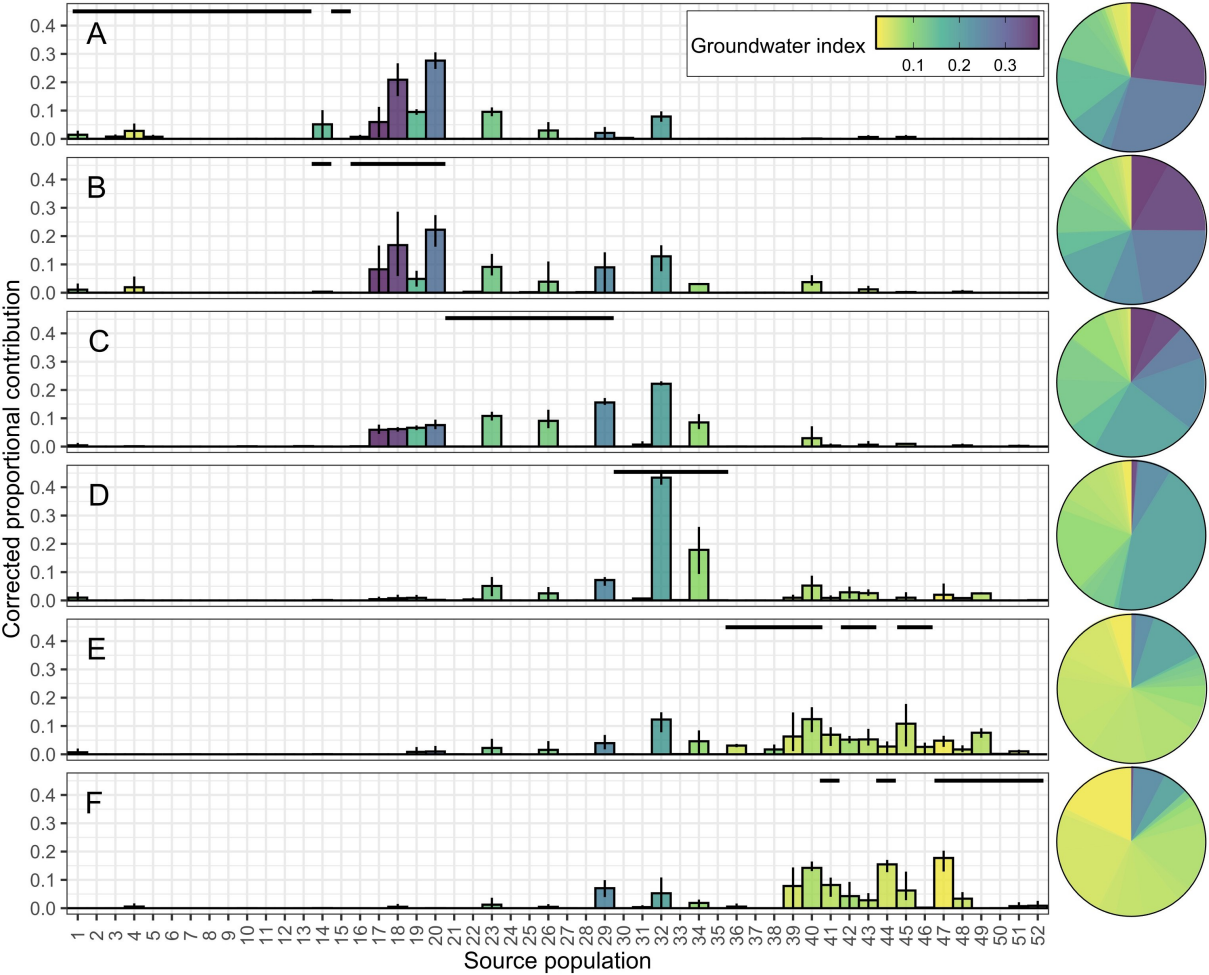
Bootstrap‐corrected proportional contribution of 52 tributary source populations to six sections of the main stem Snake River (panels). Source populations are ordered by latitude from north to south; sections are ordered from upstream to downstream (refer to numbering and lettering schemes in Figure 2). Bar height and error bars represent the contribution mean and range across 3 years of sampling (2020–2022). Bar color represents the groundwater index value calculated for each source population. Horizontal black lines denote the tributaries draining into each main stem river section. Pie charts summarize the (mean) composition of the main stem Snake River with respect to groundwater availability in the contributing source tributaries (color).

Visual inspection of bootstrap‐corrected mixing proportions suggested strong spatial structuring of tributary contributions: Upstream sections of the Snake River received contributions from more northerly source populations with high groundwater index values whereas downstream sections received contributions from more southerly source populations with lower groundwater index values (Figure 4). Model selection provided the strongest support for our second hypothesis, that population diversity varied among sections but not among years (Table 1). Although models representing spatiotemporal variability were ranked second and third (hypotheses 4 and 5, respectively), neither performed as well as the top‐ranked model.

### Drivers of tributary contributions

**FIGURE 5:**
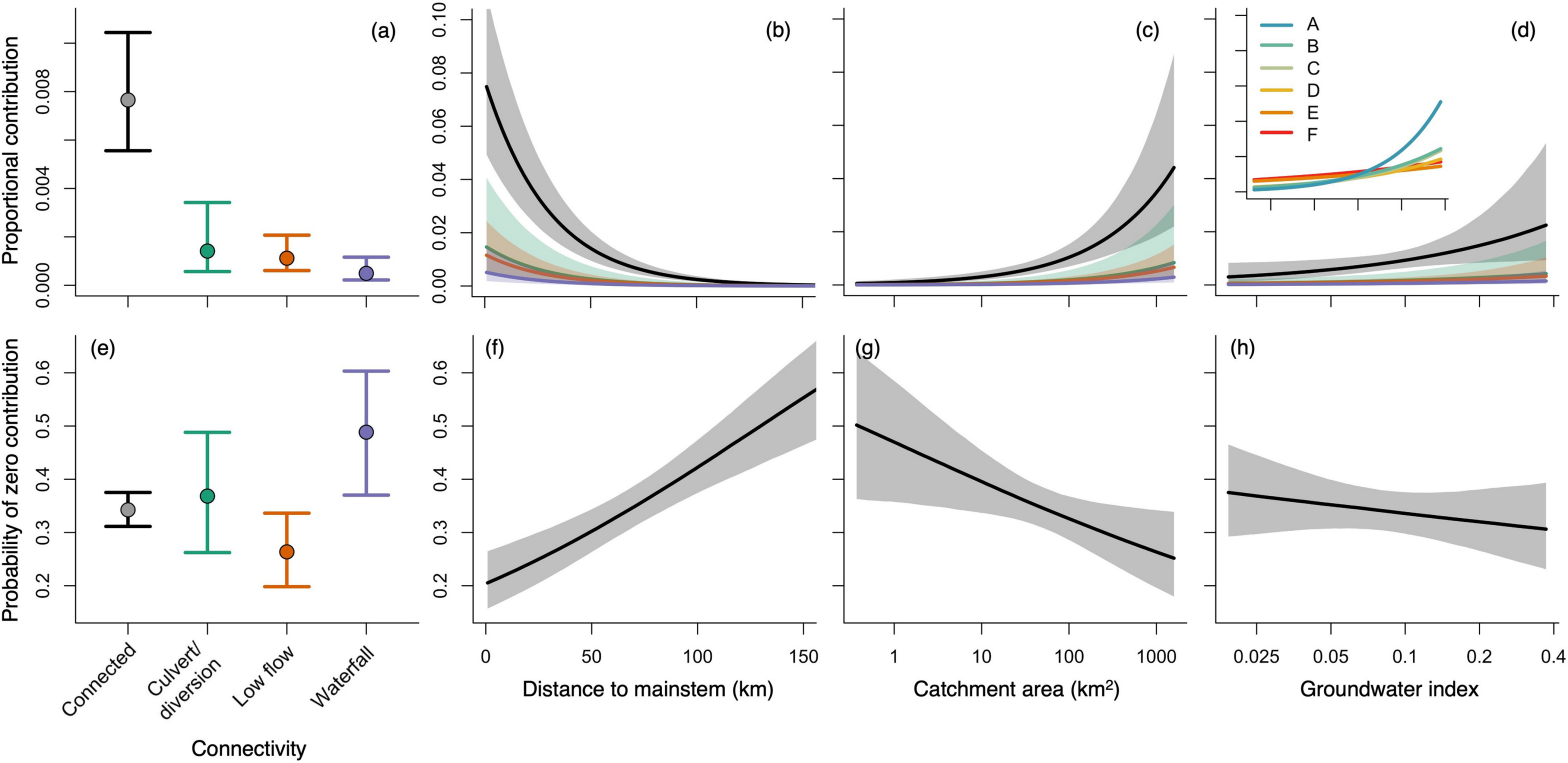
Effects of connectivity, distance to the main stem, catchment area, and groundwater on (a–d) proportional contribution and (e–h) probability of zero contribution of source populations to the main stem Snake River. Points and error bars in panels (a) and (e) and lines and shaded regions in panels (b–d) and (f–h) denote medians and 95% credible intervals, respectively. In panels (a–d), the effects of connectivity are displayed as marginal effects (with all other variables held at their mean value), whereas the effects of distance, area, and groundwater are plotted conditional on connectivity, with line color corresponding to the connectivity categories specified in panel (a). In panels (f–h), effects are shown for “connected” source populations only, as the probability of observing zero contribution did not differ among connectivity categories. Inset plot in panel (d) shows how the effect of groundwater varies among sections of the main stem Snake River (plotted for “connected” tributaries only; axis limits are the same as in the main panel).

The zero‐inflated beta regression model describing the effects of distance, area, groundwater, and connectivity on source population contributions to the main stem Snake River performed well based on posterior predictive checks (Appendix S1: Figures S6–S9). Proportional contributions from connected tributaries were approximately four to five times greater than those from disconnected tributaries, with waterfall barriers having the greatest negative effect on contributions (Figure 5a). Connectivity modulated the effects of all other covariates (additive on the logit scale). Distance had the largest effect on proportional contribution, with contributions declining as distance from the main stem increased (Figure 5b). Catchment area and groundwater had moderate and smaller positive effects on contribution, respectively (Figure 5c,d). However, the effect of groundwater varied among main stem sections, with stronger positive effects further upstream (Figure 5d, inset).

The probability of zero contribution from source populations to the main stem Snake River increased for more distant and smaller source populations (Figure 5f,g). The probability of zero contribution did not differ among connectivity categories or by groundwater index, as the 95% credible intervals for these parameters overlapped 0 (Figure 5e,h). Model precision was lowest for connected tributaries and increased for other connectivity categories (Appendix S1: Figure S10). In addition, precision increased with distance and decreased with catchment area and groundwater index.

### Spatial structure in mean contribution

**FIGURE 6:**
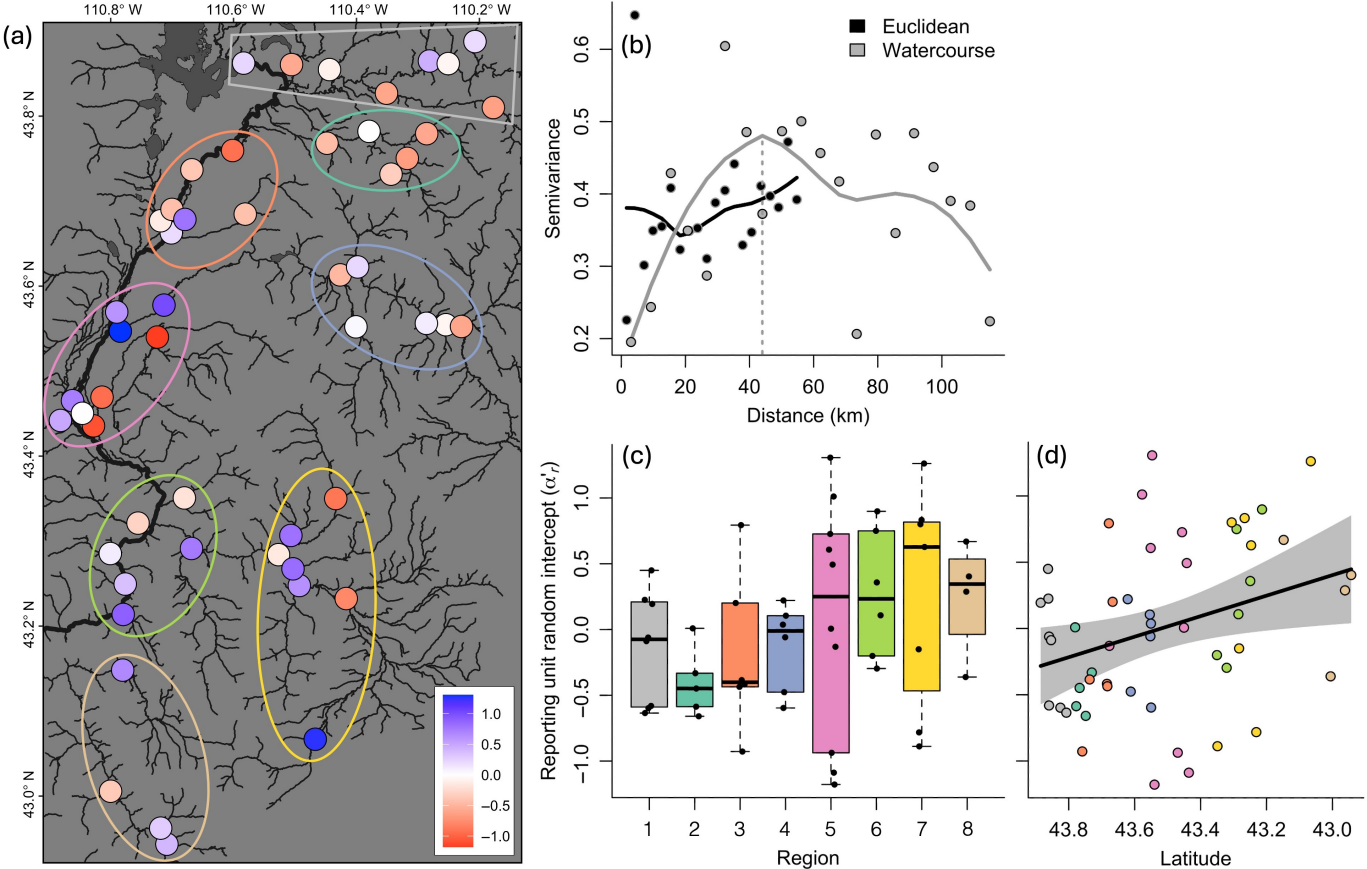
(a) Spatial pattern in tributary random intercepts: mean proportional contribution after accounting for the effects of distance, area, groundwater, and connectivity. Red and blue points mark tributaries contributing less and more than expected, respectively. Colored ovals represent regional groupings. (b) Empirical semivariogram describing spatial structure in random intercept values over Euclidean and watercourse distances. Solid lines are smoothed to data using locally estimated scatterplot smoothing (span = 0.7); vertical dashed line indicates the fine‐scale range visually determined by the primary inflection point. Relationship between source population random intercepts and (c) regional grouping and (d) latitude. Line and polygon in panel (d) represent linear regression and 95% CI, respectively.

Mean source population contribution to the main stem Snake River population of trout showed multiscale spatial structure with respect to watercourse and Euclidean distance measures (Figure 6a and Appendix S1: Figure S11). An inflection point at approximately 45 km watercourse distance indicated fine‐scale patchiness in mean contribution: Source populations located within the same region of the river network (e.g., sub‐watershed) contributed more similarly than those located within different regions (Figure 6b,c). This patchiness was superimposed over broadscale spatial dependence as suggested by a positive relationship between semivariance and Euclidean distance, reflecting a latitudinal gradient in mean contribution (Figure 6b,d). Semivariance tended to decline at far watercourse distances, but this pattern was largely driven by a single distance category.

## DISCUSSION

Population diversity is known to buffer against environmental stochasticity (e.g., Schindler et al., 2010), but less is understood about how habitat size, spatial arrangement, and local conditions shape diversity at broader scales—information vital for effective conservation and management. Using genetic stock identification, we assessed the role of tributary streams in supporting a main stem population of cold‐water fish in a high‐priority conservation area (Al‐Chokhachy et al., 2018). The main stem Snake River depended on demographic support from tributaries, but population diversity varied spatially and reflected contributions from nearby source habitats (Figure 4). Larger tributary catchments and greater groundwater availability increased contributions to the main stem, while natural and anthropogenic barriers reduced them (Figure 5). Our results highlight the reciprocal importance of main stem rivers and tributary streams to migratory fishes and illustrate how landscape structure and habitat heterogeneity shape population diversity at larger scales, providing critical guidance for conservation planning under global change.

### Migration and reciprocal connectivity in river networks

Migrants from tributary source populations accounted for 93.5% of adult trout captured in the main stem Snake River, with individuals traveling up to 134 km to access main stem habitat (Figures 3 and 4). These results highlight the importance of connected, complementary habitats, where animals move across landscapes to negotiate spatially and temporally shifting resource availability (Schlosser, 1991; van Moorter et al., 2013). Migration between seasonal ranges allows many taxa, including birds and herbivorous ungulates, to balance fitness trade‐offs between forage availability and environmental stress (Fryxell & Sinclair, 1988; Somveille et al., 2015). For riverine fishes like Yellowstone cutthroat trout, headwater tributaries provide high‐quality summer spawning habitat but harsh overwintering conditions (e.g., low flows and ice formation; Cunjak, 1996), while main stem rivers offer warmer temperatures and abundant food that enhance winter growth (Armstrong et al., 2021; Huntsman et al., 2016). Thus, migration between tributary and main stem habitats may allow fish to navigate trade‐offs arising from asynchronous resource availability.

Thirty‐six tributary source populations contributed to the main stem Snake River, supporting high trout population diversity (Figure 4). This suggests that this population may be resilient to environmental stochasticity, as increased source population richness is associated with reduced temporal variability in aggregate abundance (Carlson & Satterthwaite, 2011; Moore et al., 2021). But diversity varied spatially, with changes in the composition, richness, and evenness of source populations among river sections. This spatial heterogeneity indicates the strength of portfolio effects—and the resulting stability of the aggregate population—may differ longitudinally along the main stem (Moore et al., 2021). These results demonstrate how processes interact across spatial scales to generate emergent properties in large river ecosystems (e.g., portfolio effects; McCluney et al., 2014; Schindler et al., 2010).

While our study considers demographic contributions of tributaries to a main stem river, these results similarly suggest the value of main stem habitat to tributary populations, as trout originating from tributaries migrated long distances to access the main stem. Ultimately, resilience and productivity emerge from the dynamic, two‐way exchange of individuals and energy across the river network (Moore, 2015). Access to main stem habitats enables fish to attain larger body sizes than would be possible in headwaters alone (Al‐Chokhachy et al., 2022; Armstrong et al., 2021). Larger, highly fecund individuals may play a disproportionately large role in supporting recruitment and buffering against population declines, but this has not been tested. Headwater tributaries are key sources of individuals; but without access to main stem river habitats that alleviate physiological constraints, their broader capacity to sustain large, productive fish populations—both in the main stem and locally in tributaries—may be limited (sensu Howe et al., 1991).

Although main stem rivers can serve important rearing and growth functions (Armstrong et al., 2021; Schlosser, 1991), the high proportion of fish originating from tributaries suggests the main stem Snake River may function as a demographic sink, where persistence depends on continued immigration from tributary sources (Dias, 1996; Pulliam, 1988). Although Homel et al. (2015) estimated that 22%–30% of trout captured in the main stem Snake River spawn locally, these individuals may be strays from nearby source populations and experience limited reproductive success. Many of the locations identified as spawning habitat are unlikely to support successful reproduction because flow regulation from an upstream dam prolongs main stem riverbed mobility throughout summer egg incubation and dewaters side channels during larval emergence later in the autumn (Annear, 1989; Erwin et al., 2011; Montgomery et al., 1999). However, studies in unregulated river systems also show ubiquitous spawning migrations from main stem rivers into tributaries by cutthroat and other trout species (Muhlfeld et al., 2009; Starcevich et al., 2012). The predominance of tributary‐origin individuals may therefore reflect natural controls on the spatial distribution of adult spawning and juvenile rearing habitat (Finstad et al., 2009; Miller et al., 2008). Quantifying the availability of main stem spawning habitat, the reproductive success of individuals using these areas, and their genetic relatedness to nearby source populations would help determine whether main stem spawning constitutes a viable life history strategy.

### Drivers of spatial population diversity

Spatial shifts in the tributaries contributing to the main stem trout population offer insight into the mechanisms shaping population diversity (Figure 4). The spatial patterns are consistent with the negative effect of distance on contributions (Figure 5b). van Moorter et al. (2013) showed that individual differences in moose (*Alces alces*) migration distance were associated with the spatial scale of environmental heterogeneity. Our results support this proposed link between scales of movement and scales of change in resource profitability, as adult trout migration likely balances the cost of movement with the benefits of accessing more profitable habitat for spawning (tributaries) versus rearing (main stem rivers). However, many individuals migrated considerable distances to access main stem habitat (Figure 3), suggesting other factors may influence spatial population structure, such as competitive interactions that mediate habitat selection (sensu Fretwell & Lucas, 1970). Contributions to the main stem also increased with catchment area (Figure 5c), aligning with previous findings that larger habitats support more productive animal populations (Isaak et al., 2022; Matter, 2000), likely driven by greater resource availability. Our results are therefore consistent with spatial metapopulation theory, suggesting that population composition in shared habitats is largely determined by the spatial arrangement and size of source habitat patches (Campbell Grant et al., 2007; Moilanen & Hanski, 1998).

Although tributary distance and size influenced contributions to the main stem river, interactions between dispersal and local habitat productivity, often mediated by density dependence, can also shape spatial population structure (Pulliam & Danielson, 1991). Groundwater availability had a positive effect on tributary contributions (Figure 5d), aligning with previous findings that groundwater‐fed streams are hotspots for juvenile production with markedly higher densities (Baldock et al., 2025). Density‐dependent resource limitation during the juvenile period may promote migratory behavior (Einum et al., 2006), enhancing the representation of certain tributaries in main stem habitats. Consequently, the profitability of migration to main stem rivers may be greatest for fish originating from habitats where density‐dependent physiological constraints are most pronounced, such as groundwater‐dominated tributaries.

Ultimately, the effects of distance, catchment size, and groundwater availability were moderated by habitat connectivity. Natural and anthropogenic barriers reduced tributary contributions to the main stem Snake River (Figure 5a), consistent with known demographic and genetic impacts (Letcher et al., 2007; White et al., 2020). Nonetheless, some isolated source populations made small but potentially important contributions to the main stem. Isolated populations exhibited strong genetic differentiation from other populations with less impeded gene flow (Appendix S1: Figure S2; Wofford et al., 2005). Therefore, even limited input from such sources may help maintain genetic diversity in downstream habitats, a key factor in adaptation to global climate change (Walsworth et al., 2019).

Multiscale spatial structure in tributary random intercepts (Figure 6) suggests untested but spatially autocorrelated factors likely influence demographic contributions to the main stem. We observed a latitudinal gradient in random intercepts, with southerly tributaries contributing more than northerly tributaries—after accounting for distance, catchment area, groundwater, and connectivity. This pattern may reflect broadscale gradients in physicochemical conditions (e.g., geology and nutrient availability) and associated effects on food web productivity (Poff & Huryn, 1998). Random intercepts also clustered regionally, suggesting that shared environmental or genetic factors create hotspots of highly productive tributaries. A better understanding of genetic population structure may clarify the mechanisms driving this spatial pattern. Likewise, understanding whether genetic diversity of tributary populations scales with catchment size, and quantifying the effect of tributary contributions on main stem genetic diversity could provide insight into the effects of tributaries on adaptive capacity and resilience of main stem river fish populations under climate change. Collectively, our results highlight how landscape structure, habitat heterogeneity, and evolutionary processes interact across scales to generate and maintain population diversity in large river networks (McCluney et al., 2014) and point to future work in population genetics that could further elucidate these patterns.

### Riverscape conservation under global change

Spatially explicit data are essential for conservation planning but can be challenging to obtain for species with broad habitat use. Molecular tools such as genetic stock identification offer a solution as technological advances lower barriers to integrating genetic data into management decisions (Meek & Larson, 2019; Scribner et al., 2016). For example, genetic stock identification can expand the coverage of traditional monitoring programs by detecting contributions from unmonitored tributaries and assessing how these vary over time. While we observed little temporal variation over 3 years (Table 1), monitoring may reveal asynchronous tributary contributions over longer timescales, indicative of portfolio effects (Schindler et al., 2010). Furthermore, genetic stock identification may help prioritize habitat protection efforts (e.g., of groundwater‐fed tributaries) and detect the re‐emergence of migratory strategies following the restoration of connectivity. As global change intensifies, adopting innovative tools to monitor species with complex life histories will be increasingly important for effective management.

One approach to cold‐water species management under climate change emphasizes protecting headwater streams (Isaak et al., 2015; Isaak & Young, 2023). When non‐native species pose additional risks, “conservation barriers” may be used to isolate native species from competitors and/or hybridization threats. While considering refugia and non‐natives remains important, focusing solely on headwaters does not fully consider the complementary ecological roles of headwater tributaries and larger main stem rivers for species whose life history needs span the riverscape (Armstrong et al., 2021; Novinger & Rahel, 2003). Our results highlight this reciprocity: Main stem rivers depend on demographic contributions from tributaries, and tributary populations use main stem habitat. Beyond their ecological importance, main stem rivers support valuable recreational fisheries (Cline et al., 2022). Thus, management plans for large rivers that do not address the ecological contributions of tributaries may not achieve optimal outcomes (Bouska et al., 2023). Similarly, conservation strategies that exclusively focus on headwater streams may fail to capture the broader habitat requirements necessary to maintain robust cold‐water fish populations and associated fishing economies (Rossi et al., 2025).

Understanding the mechanisms underlying population diversity is essential for designing conservation strategies that maintain resilience and reduce extinction risk (Anderson et al., 2015; Haak & Williams, 2012). Here, we show how tributary streams shape the demographic diversity of a main stem river population of Yellowstone cutthroat trout, a native cold‐water fish with significant ecological, economic, and cultural value. Our results demonstrate how landscape structure and habitat heterogeneity influence population diversity at broader spatial scales (Campbell Grant et al., 2007; McCluney et al., 2014). Tributaries and main stem rivers cannot be considered in isolation, but rather as components of a broader network of habitats across which individuals migrate as physiological and life history needs change (Schlosser, 1991). Ensuring the long‐term viability of river ecosystems will require protecting diverse portfolios of tributaries and maintaining connectivity to main stem habitats, especially in the face of global change (Bouska et al., 2023).

## AUTHOR CONTRIBUTIONS

Jeffrey R. Baldock and Annika Walters conceived the study, led the design of methodology, and acquired project funding. Jeffrey R. Baldock led the field sampling and investigation, led the analysis, and wrote the initial draft of the manuscript. William C. Rosenthal assisted with the analysis. Matthew R. Campbell coordinated and supervised genetic sequencing. All authors provided feedback on the study design and analysis and edited the manuscript.

## CONFLICT OF INTEREST STATEMENT

The authors declare no conflicts of interest.

## Supporting information


Appendix S1.

## Data Availability

Data (Baldock et al., 2026) are provided in a US Geological Survey data release at https://doi.org/10.5066/P134IPH5. Code (Baldock, 2026) is available in Zenodo at https://doi.org/10.5281/zenodo.18942196.
