# Supplementary material for: Riverscape heterogeneity shapes population diversity for a migratory fish
Source: Ecol Appl. 2026 Jun 5;36(4):e70247. doi: 10.1002/eap.70247 (PMC13241588; doi:10.1002/eap.70247)
Supplement: Supplementary file 1 — Appendix S1. [file EAP-36-e70247-s001.pdf]

Appendix S1

Riverscape heterogeneity shapes population diversity for a migratory fish

Jeffrey R. Baldock, William C. Rosenthal, Robert K. Al-Chokhachy, Matthew R. Campbell, Catherine E. Wagner, and Annika Walters

Ecological Applications

Disclaimer: *Any use of trade, firm, or product names is for descriptive purposes only and does not imply endorsement by the U.S. Government.*

Table S1. Source population details. Fields are defined as follows. *Code*: numeric code as specified in Figure 1. *Name* = stream name(s) as provided in the Geographic Names Information System (GNIS); additional location information provided in parentheses; italics case denotes unnamed streams in the GNIS but recognized as such locally. *Lat.*: latitude. *Long.*: longitude. N_c: number of collections. *N*: sample size (number of fish). *Self-assign.*: self-assignment rate (Figure S1). *Mean resid.*: mean residuals from simulated mixtures (mean and standard deviation, Figure S3). *Area*: catchment area in square kilometers. *GW*: groundwater index. *Connectivity*: tributary to mainstem connectivity classification.

Code	Alias	Lat.	Long.	N_c	N	Self-assign.	Mean resid.	Area	GW	Connectivity
1	North Buffalo Fork	43.885	-110.208	1	40	0.66	0.006 (0.007)	211.728	0.109	Connected
2	Box Creek	43.863	-110.282	1	16	1	-0.006 (0.006)	29.141	0.025	Low flow
3	Spring Creek (NPS)	43.862	-110.584	1	25	0.96	-0.001 (0.004)	4.013	0.092	Culvert/div.
4	Pacific Creek	43.86	-110.506	2	62	0.895	0 (0.004)	415.057	0.042	Connected
5	Clear Creek	43.859	-110.251	1	37	0.914	0.001 (0.006)	16.604	0.029	Connected
6	Lava Creek	43.853	-110.444	1	34	1	0 (0.004)	65.334	0.037	Culvert/div.
7	Blackrock Creek (lower)	43.825	-110.352	1	29	0.962	-0.002 (0.005)	124.922	0.044	Connected
8	Blackrock Creek (upper)	43.807	-110.179	1	27	1	0 (0.004)	80.686	0.069	Connected
9	Spread Creek	43.78	-110.381	1	33	0.818	0.002 (0.006)	202.609	0.045	Connected
10	North Fork Spread and Flagstaff Creeks	43.777	-110.287	2	14	0.889	-0.008 (0.007)	62.106	0.077	Connected
11	Rock Creek	43.767	-110.449	1	20	1	0 (0.004)	11.986	0.019	Connected
12	*Deadmans Bar Spring*	43.759	-110.603	1	25	0.955	-0.003 (0.005)	11.761	0.123	Connected
13	South Fork Spread Creek	43.748	-110.319	1	36	0.895	0.003 (0.006)	98.903	0.033	Connected
14	*Cowboy Cabin Spring*	43.736	-110.669	2	23	0.692	-0.013 (0.008)	6.639	0.151	Connected
15	Leidy Creek	43.73	-110.346	1	16	1	0 (0.004)	10.736	0.045	Connected
16	Ditch Creek	43.684	-110.583	1	23	1	0 (0.004)	67.691	0.038	Low flow
17	*Snake River Side Channel Spring*	43.683	-110.701	1	31	0.667	-0.003 (0.006)	18.81	0.372	Connected
18	*Upper Bar BC Spring*	43.678	-110.705	1	39	0.619	0.004 (0.007)	5.737	0.352	Connected
19	Cottonwood Creek (NPS)	43.677	-110.707	1	40	0.796	0.007 (0.006)	187.153	0.152	Connected
20	*Blacktail Spring*	43.667	-110.703	1	42	0.559	0.017 (0.011)	61.145	0.266	Connected
21	Slate Creek	43.621	-110.401	2	34	1	-0.001 (0.003)	97.257	0.033	Waterfall
22	Crystal Creek (lower)	43.612	-110.429	1	30	0.862	0.002 (0.006)	185.115	0.053	Low flow
23	Gros Ventre River (lower)	43.577	-110.715	1	38	0.571	0.013 (0.007)	1589.647	0.128	Low flow
24	Goosewing Creek	43.554	-110.29	1	10	0.833	-0.009 (0.009)	40.396	0.045	Low flow
25	Cottonwood Creek (Gros Ventre basin)	43.552	-110.259	1	34	0.892	0.006 (0.007)	89.178	0.074	Low flow
26	*Three Channel Spring*	43.551	-110.791	2	39	0.667	-0.005 (0.006)	32.365	0.118	Connected
27	Crystal Creek (upper)	43.551	-110.404	1	29	0.839	0.001 (0.005)	155.612	0.035	Waterfall
28	Fish Creek (Gros Ventre basin)	43.55	-110.246	1	17	0.917	-0.005 (0.006)	587.876	0.057	Low flow
29	*Lower Bar BC Spring*	43.547	-110.786	1	40	0.574	0.005 (0.007)	6.397	0.238	Connected
30	Flat Creek	43.54	-110.726	1	30	1	-0.003 (0.005)	173.369	0.237	Connected
31	Spring Creek (TSS)	43.469	-110.815	1	15	1	-0.003 (0.004)	24.858	0.2	Connected
32	Fish Creek (Wilson)	43.456	-110.863	2	82	0.68	0.017 (0.006)	233.374	0.193	Connected
33	*Ford Spring*	43.45	-110.847	1	13	0.75	-0.01 (0.008)	0.371	0.308	Connected
34	Mosquito Creek	43.442	-110.883	1	40	0.865	-0.003 (0.004)	61.641	0.088	Connected
35	Cody and Blue Crane Creeks	43.436	-110.829	2	18	1	-0.001 (0.003)	18.512	0.297	Connected
36	Horse Creek	43.35	-110.683	1	24	1	-0.002 (0.004)	63.204	0.054	Culvert/div.
37	Granite Creek (upper)	43.349	-110.439	1	17	1	-0.006 (0.006)	131.175	0.051	Connected
38	Fall and Coburn Creeks	43.321	-110.758	3	75	1	0 (0.008)	116.532	0.106	Waterfall
39	Boulder Creek	43.306	-110.512	1	29	0.8	-0.001 (0.005)	53.7	0.05	Connected
40	Willow Creek	43.291	-110.672	1	40	0.483	0.015 (0.008)	185.703	0.072	Connected
41	Dog Creek	43.285	-110.803	1	46	0.953	0 (0.005)	31.09	0.062	Connected
42	Granite Creek (lower)	43.283	-110.532	1	38	0.842	0.001 (0.005)	220.383	0.079	Connected
43	Shoal Creek	43.266	-110.509	1	40	0.795	0.002 (0.005)	82.65	0.066	Connected
44	Cabin Creek	43.249	-110.779	1	37	0.816	0.001 (0.005)	23.51	0.05	Connected
45	Cliff Creek	43.247	-110.499	1	38	0.81	0.003 (0.005)	158.381	0.06	Connected
46	Dell Creek	43.231	-110.423	1	31	1	-0.003 (0.005)	118.157	0.051	Connected
47	Bailey Creek	43.214	-110.782	1	29	0.737	-0.009 (0.008)	41.571	0.028	Connected
48	Little Greys River and Steer Creek	43.148	-110.783	3	77	0.939	0.004 (0.005)	179.346	0.057	Connected
49	Hoback River (upper)	43.066	-110.474	1	38	0.895	0.001 (0.005)	114.078	0.067	Connected
50	White Creek	43.006	-110.803	1	14	1	-0.009 (0.008)	32.672	0.064	Connected
51	Deadman Creek (Greys)	42.962	-110.723	1	26	0.864	-0.003 (0.006)	42.607	0.042	Connected
52	Blind Bull Creek	42.943	-110.713	1	30	0.821	0.001 (0.006)	36.352	0.07	Connected

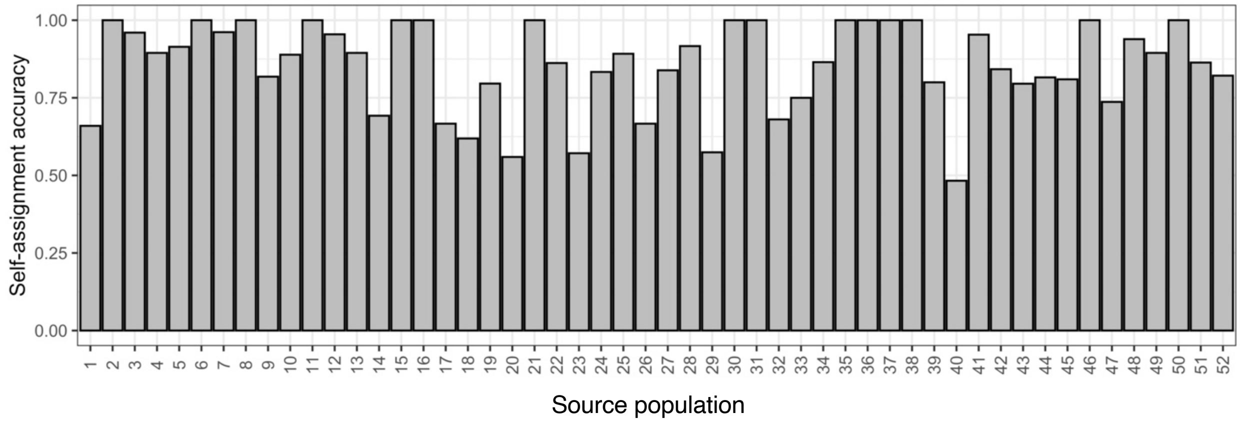

Figure S1. Self-assignment rates for each source population (numbered as in Table S1) obtained from the "self_assign" function in the R package *rubias* (Moran and Anderson 2019). Self-assignment rates refer to the proportion of individuals that correctly assigned to their reporting unit of origin.

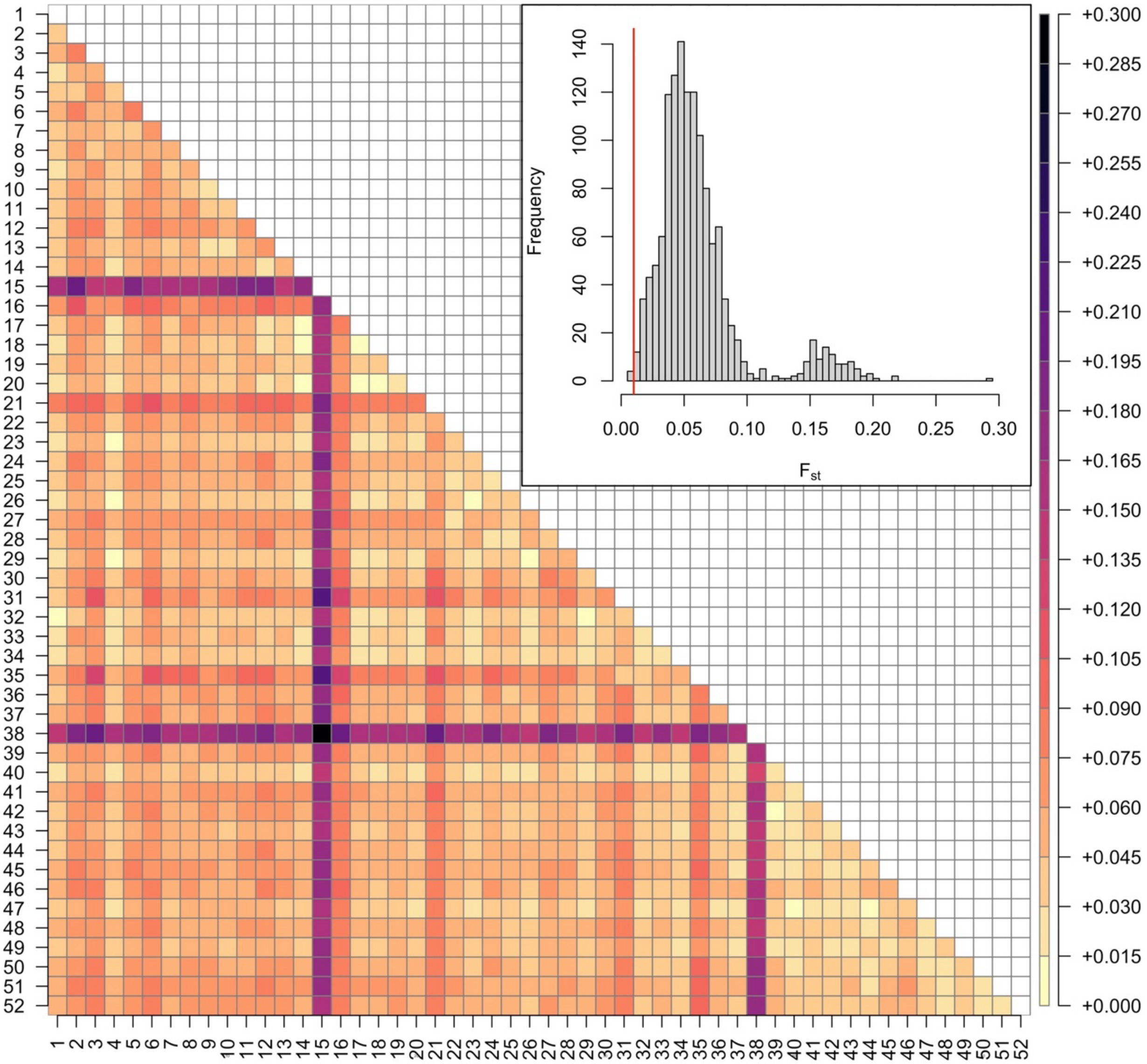

Figure S2. Pairwise measures of genetic differentiation among source populations (refer to Table S1 for names), F_{st} (Weir and Cockerham 1984). Inset histogram shows the distribution of pairwise F_{st} values, with the minimum recommended value for accurate genetic stock identification (0.01; Araujo et al. 2014) denoted by the vertical red line.

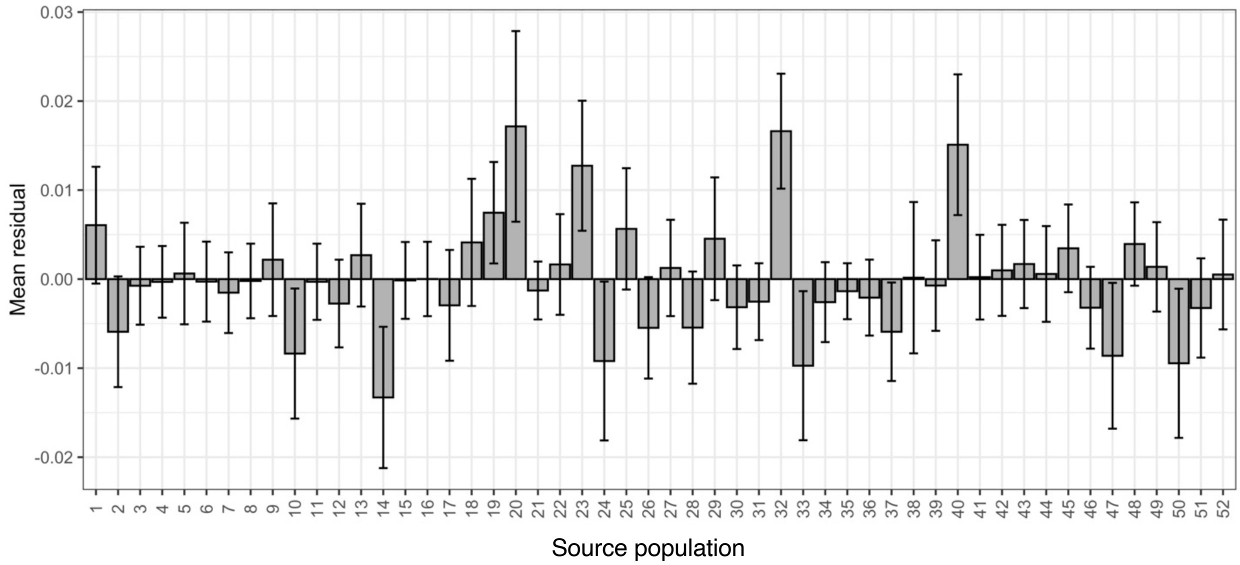

Figure S3. Source population-specific mean residuals from 500 simulated mixtures calculated using the "assess_reference_loo" function in the R package *rubias* (Moran and Anderson 2019). *Error bars* represent standard deviations. Refer to Table S1 for source population names.

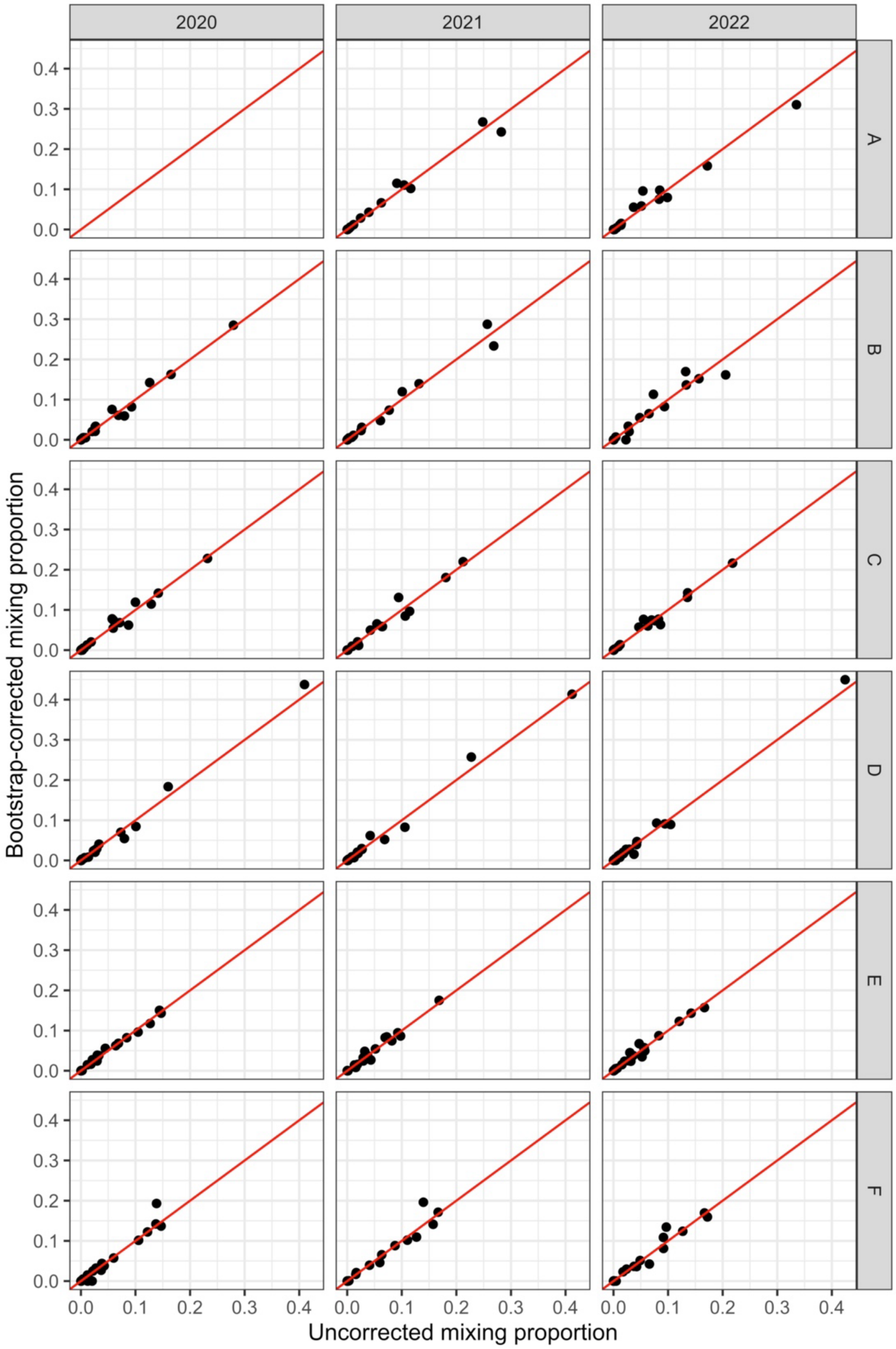

Figure S4. Relationship between uncorrected and bootstrap-corrected mixing proportions across six sections of the Snake River (*rows*) and three years (*columns*). *Red lines* represent 1:1 relationships.

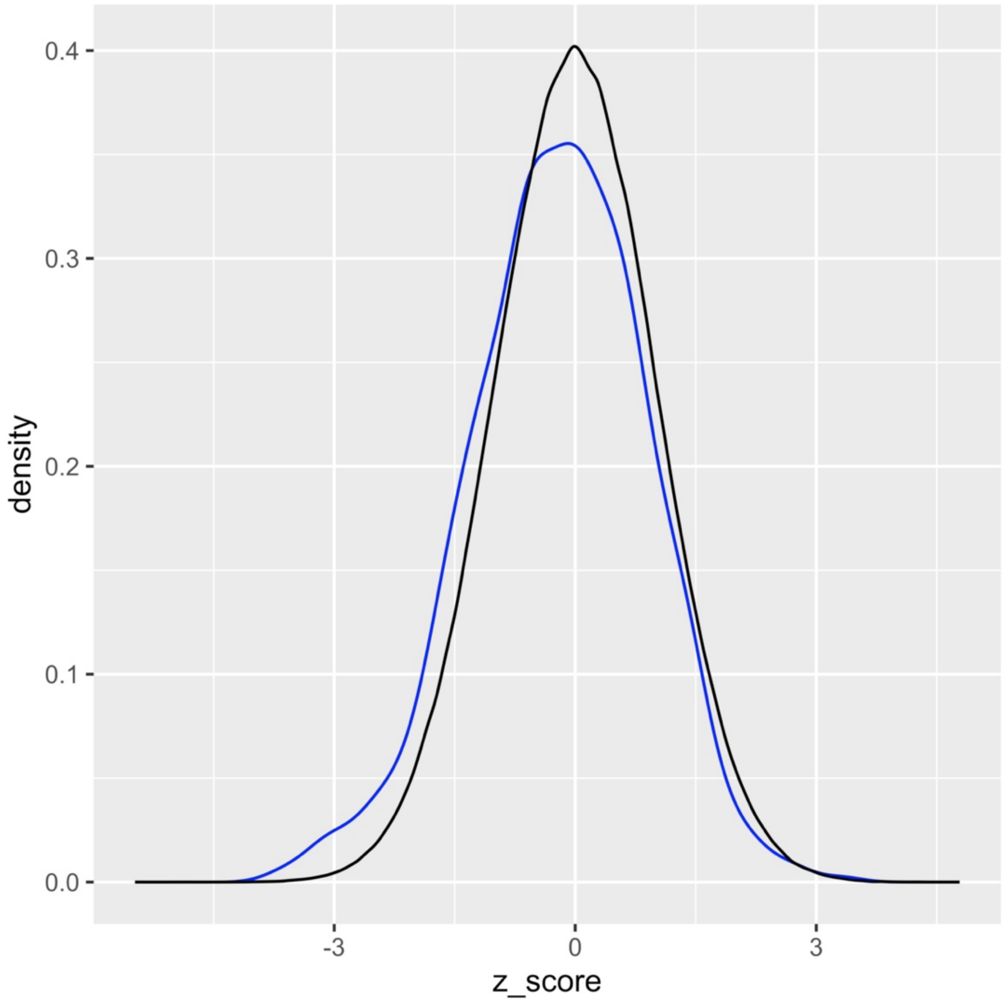

Figure S5. Distribution of z-scores (*blue line*) relative to a normal distribution (*black line*; expected outcome if all mixture samples assigned with high certainty to source populations in the baseline dataset).

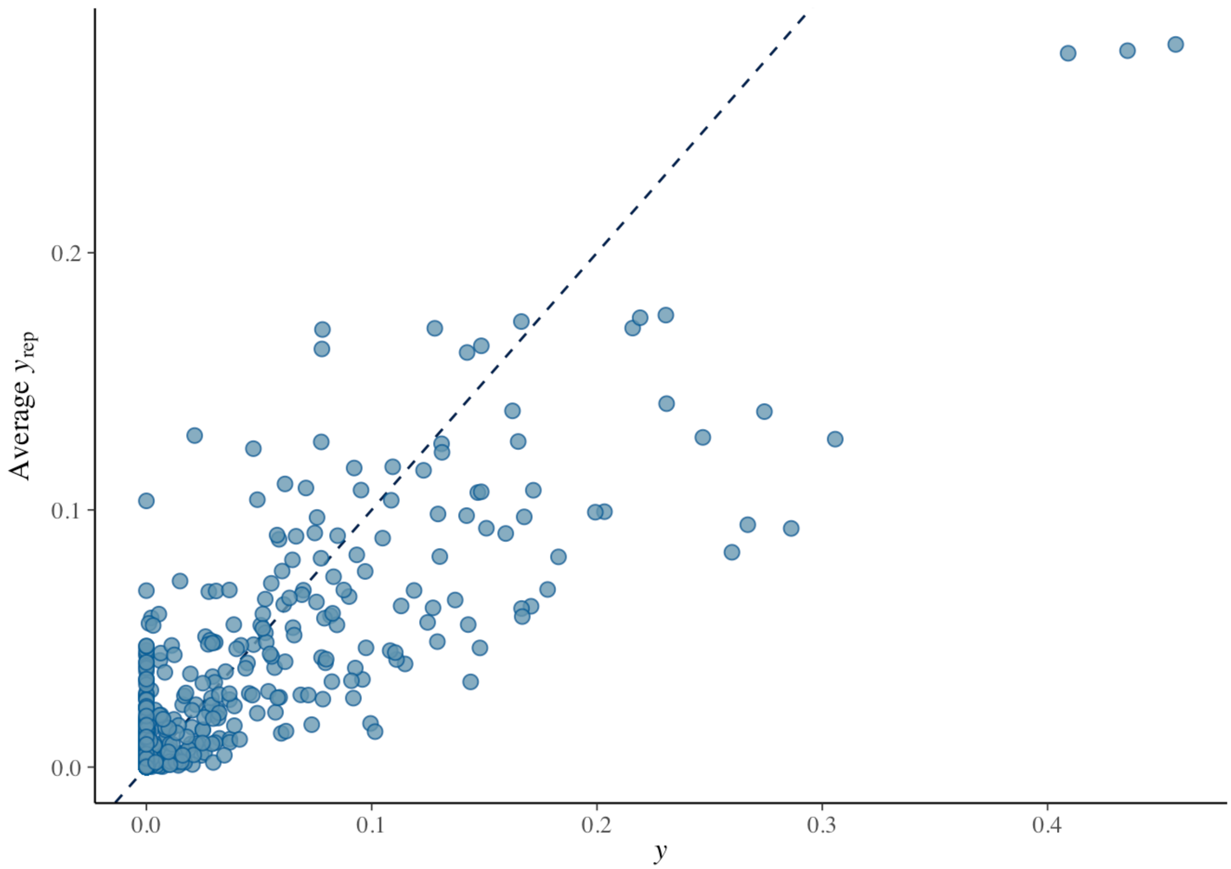

Figure S6. Beta regression posterior predictive check showing the relationship between model predictions ("Average y_{rep}") and observed data ("y"). *Dashed line* represents the 1:1 line.

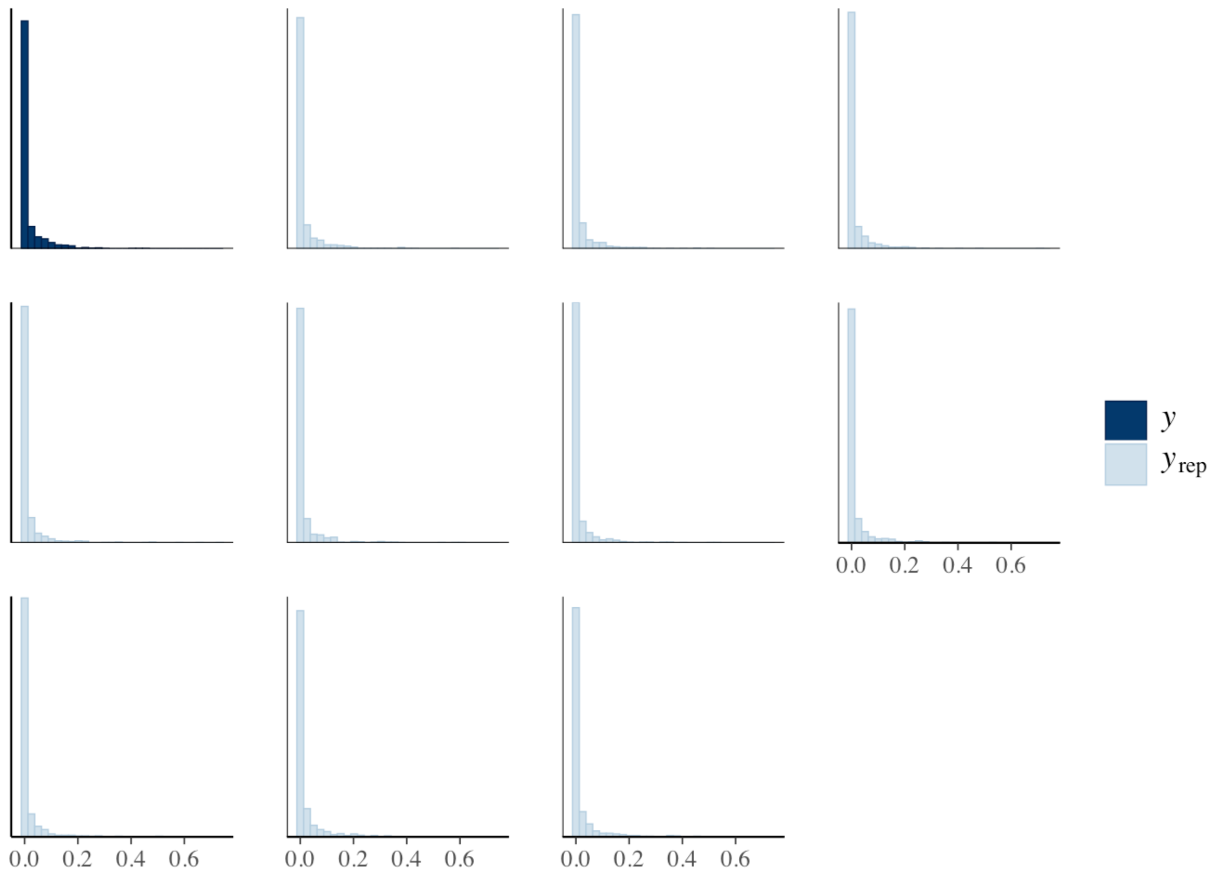

Figure S7. Beta regression posterior predictive check showing the distribution of the observed data (“y”, top-left panel) and model predictions (“y_{rep}”) for 10 MCMC iterations (all other panels).

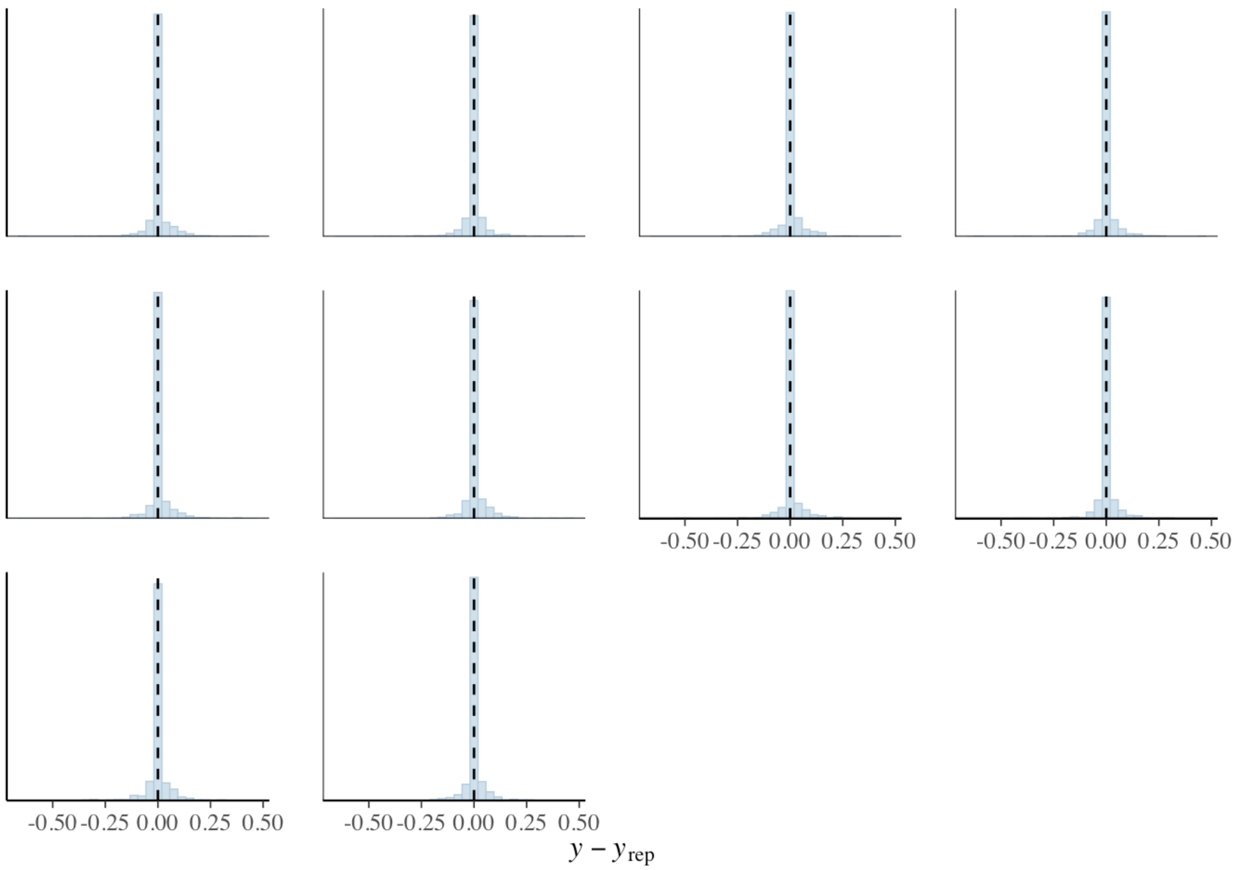

Figure S8. Beta regression posterior predictive check showing the distribution of residuals ("y - y_{rep}") for 10 MCMC iterations (*panels*).

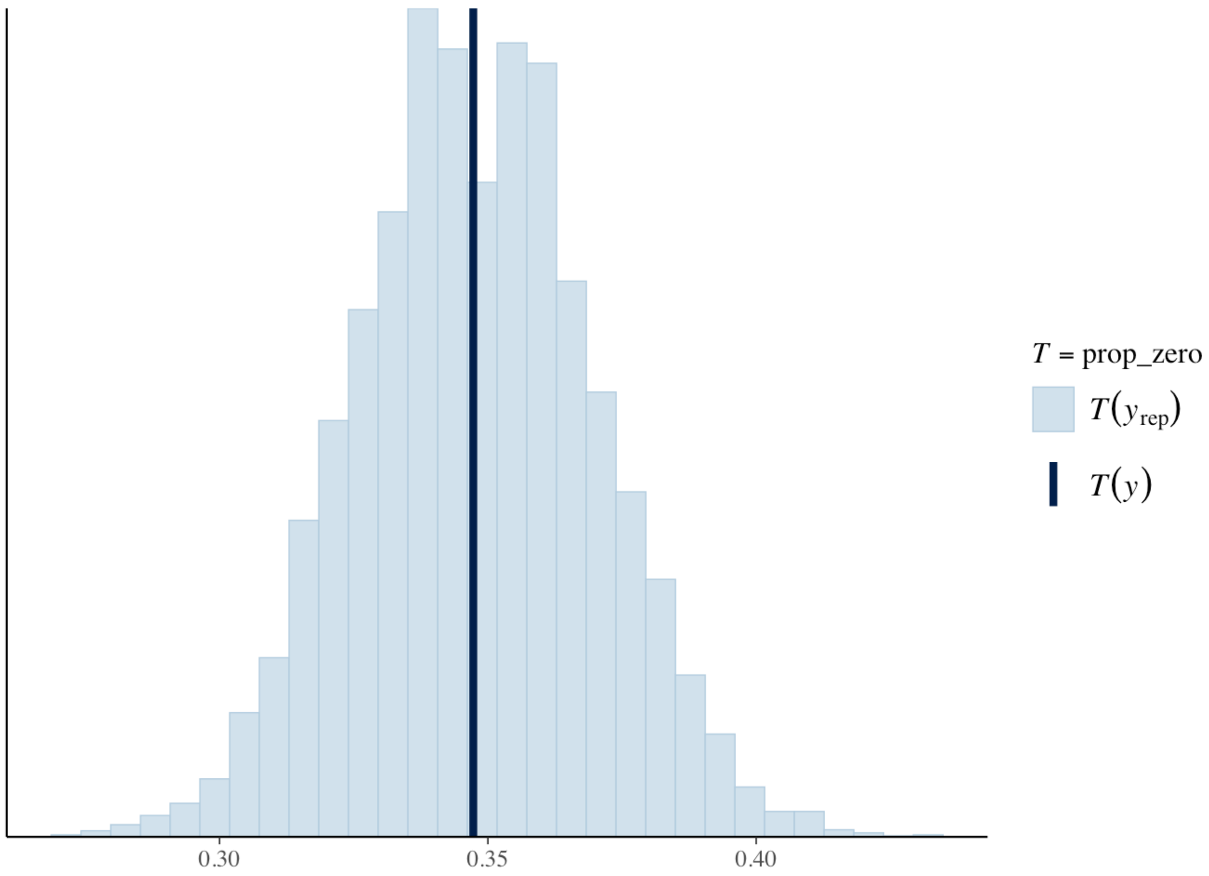

Figure S9. Beta regression posterior predictive check showing the proportion of 0s in the observed data (*horizontal dark line*) compared to the proportion of 0s in the model predictions, across all MCMC iterations (*histogram*).

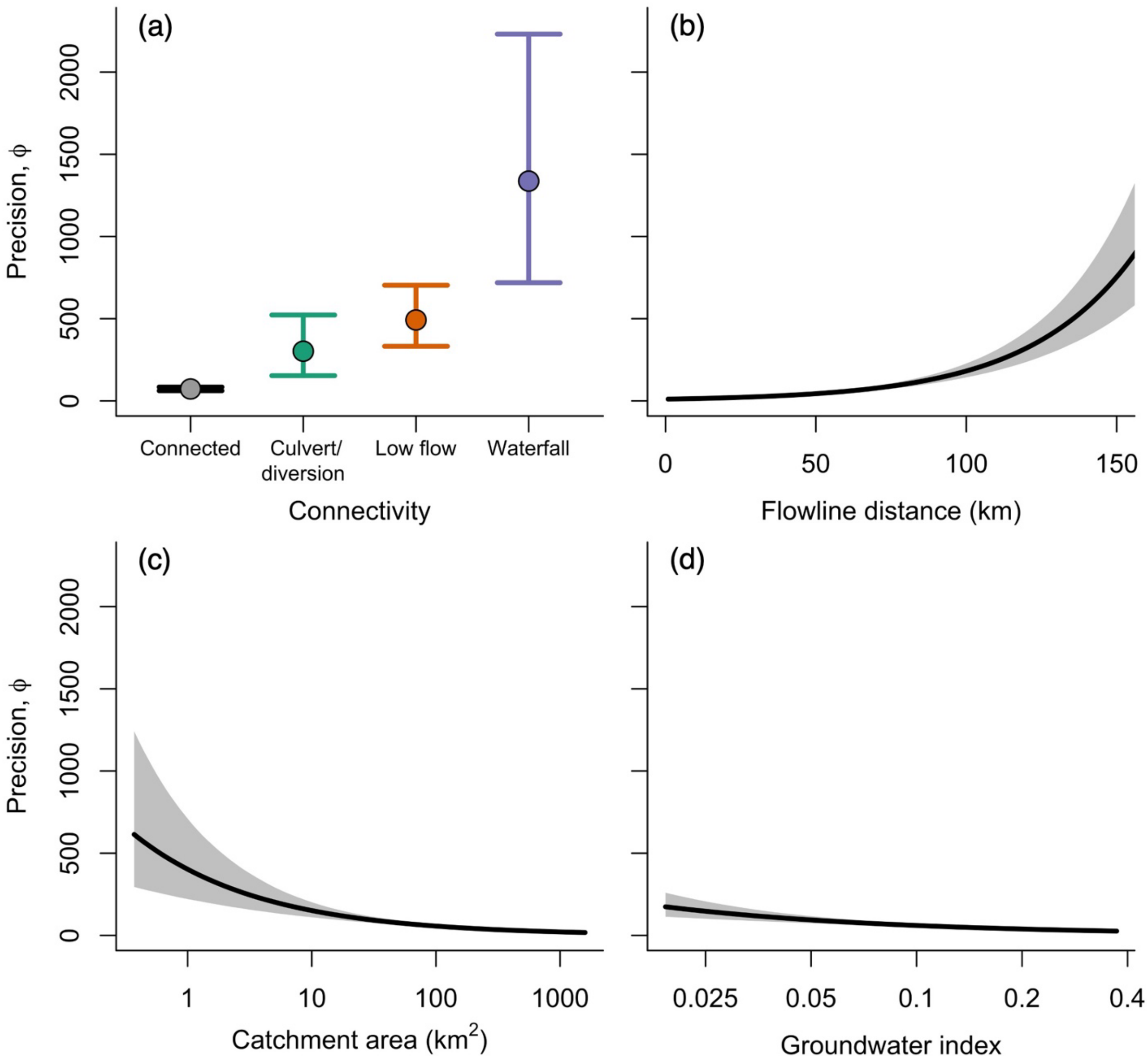

Figure S10. Marginal effects of (a) connectivity, (b) flowline distance (km), (c) catchment area (km^2), and (d) groundwater index on precision of the beta regression model. Marginal effects of distance, area, and groundwater are shown for "connected" reporting groups only.

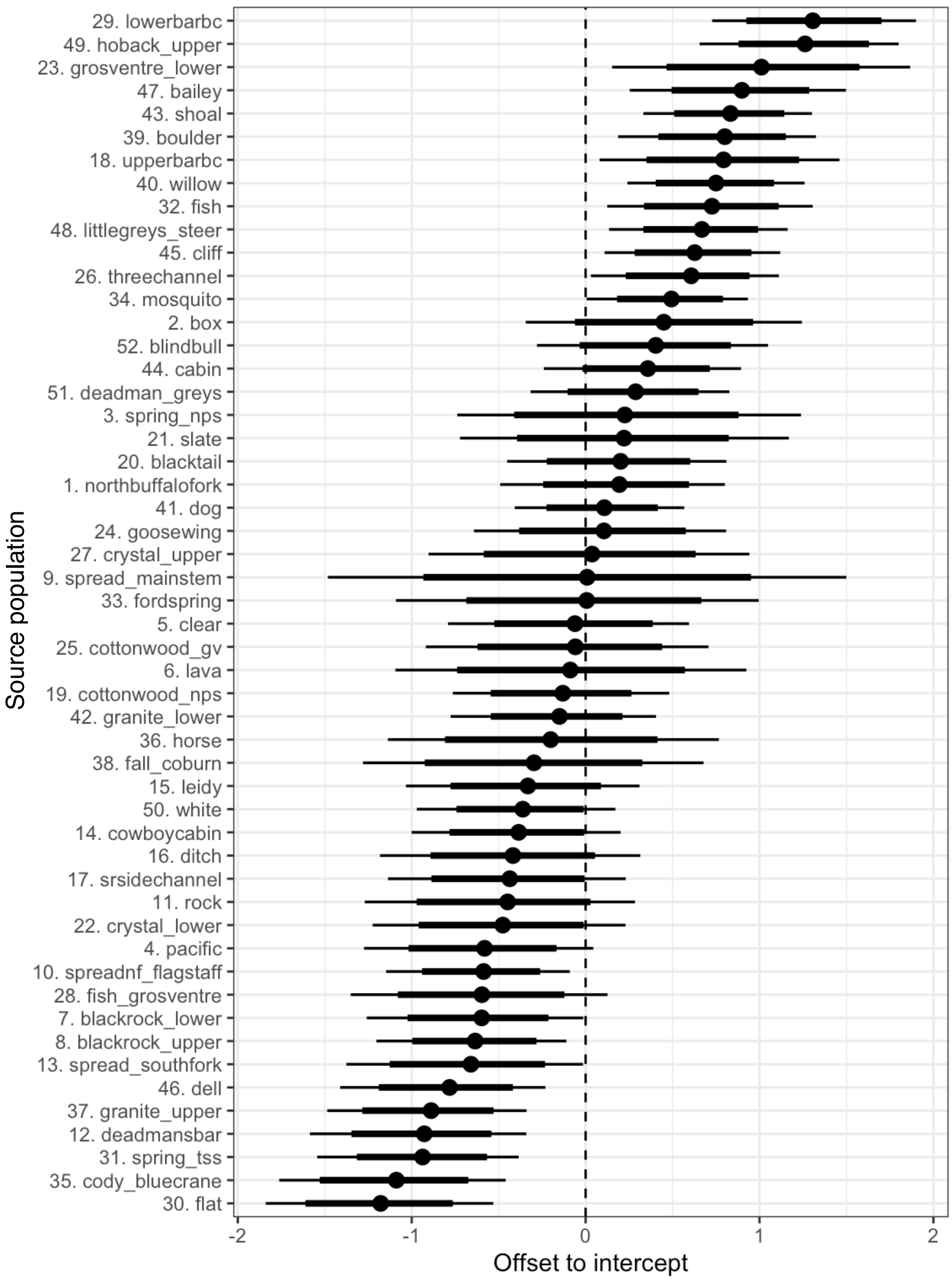

Figure S11. Beta regression model posterior predicted random intercepts for source population, α_r' (estimated as offsets to the global intercept). Random intercepts represent the mean source population proportional contribution after accounting for the main effects of distance, catchment area, groundwater availability, and connectivity. Points represent posterior medians and thick and thin horizontal error bars represent the 80% and 95% credible intervals, respectively.

References:

Moran, B. M., and E. C. Anderson. 2019. Bayesian inference from the conditional genetic stock identification model. Canadian Journal of Fisheries and Aquatic Sciences 76:551–560.
